# The role of clonal communication and heterogeneity in breast cancer

**DOI:** 10.1186/s12885-019-5883-y

**Published:** 2019-07-05

**Authors:** Ana Martín-Pardillos, Ángeles Valls Chiva, Gemma Bande Vargas, Pablo Hurtado Blanco, Roberto Piñeiro Cid, Pedro J. Guijarro, Stefan Hümmer, Eva Bejar Serrano, Aitor Rodriguez-Casanova, Ángel Diaz-Lagares, Josep Castellvi, Samuel Miravet-Verde, Luis Serrano, María Lluch-Senar, Víctor Sebastian, Ana Bribian, Laura López-Mascaraque, Rafael López-López, Santiago Ramón y Cajal

**Affiliations:** 1 Translational Molecular Pathology Group, Vall d’Hebron Research Institute, Barcelona, Spain; 2 CIBERONC (Centro de Investigación Biomédica en Red de Cáncer), Madrid, Spain; 3 Cancer Modelling Lab, Roche-CHUS Joint Unit, Santiago de Compostela, Spain; 4 Cancer Epigenomics, Translational Medical Oncology (Oncomet), Health Research Institute of Santiago (IDIS), University Clinical Hospital of Santiago (CHUS), Santiago de Compostela, Spain; 5 Hospital Vall d’Hebron, Anatomía Patológica, Barcelona, Spain; 6 EMBL/CRG Systems Biology Research Unit, Centre for Genomic Regulation (CRG), The Institute of Science and Technology, Barcelona, Spain; 7 Universitat Pompeu Fabra (UPF), Barcelona, Spain; 8 Institució Catalana de Recerca i Estudis Avançats (ICREA), Barcelona, Spain; 9 Department of Chemical Engineering, Aragon Institute of Nanoscience (INA), University of Zaragoza, Zaragoza, Spain; 10 Networking Research Centre on Bioengineering, Biomaterials and Nanomedicine, CIBER-BBN, Madrid, Spain; 11 Department of Molecular, Cellular and Developmental Neurobiology, Instituto Cajal-CSIC, Madrid, Spain; 12 Roche-CHUS Joint Unit, University Clinical Hospital of Santiago (CHUS), Santiago de Compostela, Spain

**Keywords:** Tumor, Breast, Cancer, Metastasis, Heterogeneity, Clone, Communication, Cooperation, MDA-MB-231

## Abstract

**Background:**

Cancer is a rapidly evolving, multifactorial disease that accumulates numerous genetic and epigenetic alterations. This results in molecular and phenotypic heterogeneity within the tumor, the complexity of which is further amplified through specific interactions between cancer cells. We aimed to dissect the molecular mechanisms underlying the cooperation between different clones.

**Methods:**

We produced clonal cell lines derived from the MDA-MB-231 breast cancer cell line, using the UbC-StarTrack system, which allowed tracking of multiple clones by color: GFP C3, mKO E10 and Sapphire D7. Characterization of these clones was performed by growth rate, cell metabolic activity, wound healing, invasion assays and genetic and epigenetic arrays. Tumorigenicity was tested by orthotopic and intravenous injections. Clonal cooperation was evaluated by medium complementation, co-culture and co-injection assays.

**Results:**

Characterization of these clones in vitro revealed clear genetic and epigenetic differences that affected growth rate, cell metabolic activity, morphology and cytokine expression among cell lines. In vivo, all clonal cell lines were able to form tumors; however, injection of an equal mix of the different clones led to tumors with very few mKO E10 cells. Additionally, the mKO E10 clonal cell line showed a significant inability to form lung metastases. These results confirm that even in stable cell lines heterogeneity is present. In vitro*,* the complementation of growth medium with medium or exosomes from parental or clonal cell lines increased the growth rate of the other clones. Complementation assays, co-growth and co-injection of mKO E10 and GFP C3 clonal cell lines increased the efficiency of invasion and migration.

**Conclusions:**

These findings support a model where interplay between clones confers aggressiveness, and which may allow identification of the factors involved in cellular communication that could play a role in clonal cooperation and thus represent new targets for preventing tumor progression.

**Electronic supplementary material:**

The online version of this article (10.1186/s12885-019-5883-y) contains supplementary material, which is available to authorized users.

## Background

Breast cancer is the most common cancer in women worldwide and the second leading cause of cancer-related death. In breast cancer, metastatic disease progression carries a poor prognosis, with 5-year survival rates of below 20% [1, 2]. The prognosis is even worse in the case of triple negative breast cancer (TNBC), which lacks the major targets of approved therapies, limiting treatment options to surgery, radiotherapy and/or systemic chemotherapy [3].

Breast cancer, like many other malignant tumors, shows a great molecular and phenotypic heterogeneity, both at an inter- and intra-tumoral level [4–6]. This heterogeneity may be understood to be due to the accumulation of molecular alterations from an initial clone that undergoes Darwinian selection. During this evolution, certain core molecular alterations will be largely governed by cell-extrinsic phenomena, such as environmental conditions, including the immune response [7–12]. This mechanism of tumor evolution has been observed in multiple tumor types [13–15]. Tumor heterogeneity can result in an incomplete or incorrect diagnosis when small tumor samples or biopsies are used; consequently, treatments may be directed against targets that are not expressed throughout the entire tumor [16–18].

Furthermore, the genetic-based type of tumor evolution cannot fully explain how all the functional and phenotypic alterations required to fulfill Weinberg’s 10 hallmarks of cancer [19] can accumulate within a single cell clone. For this reason, the theory of clonal cooperation asserts that tumor clones have complementary genetic alterations that synergistically contribute to tumor progression and metastasis [20–27]. This clonal cooperation, together with the influence of the tumor stroma and the immune system, is more likely to give rise to a functional consortium capable of altering all the biochemical pathways required for tumor formation [20].

We therefore reason that analysis of tumor heterogeneity should not only be based on genetic alterations (mutations, amplifications, and translocations, among others) but should be complemented with functional analysis based on protein expression and pathway analysis to reveal tumor heterogeneity at the phenotypic level [15, 28–31]. Phenotypic heterogeneity may also be caused by non-genetic alterations such as epigenetic changes or factors secreted from other cells within the tumor or tumor environment [14, 18, 27, 32, 33]. In addition, conditions within the tumor, such as hypoxia, oxidative stress or starvation are not reflected in genetic alterations, even though numerous adaptive changes (e.g. metabolism) can be observed within the affected cells [28]. Since most of these changes are brought about by altered cell signaling pathways, the evaluation of expression levels and activity status (e.g. phosphorylation) of signaling factors is currently the best approach to monitor functional tumor heterogeneity [14, 28, 32, 34].

In order to study intratumoral heterogeneity and clonal cooperation on a functional level, we characterized the phenotypic features of individual clones isolated from a breast carcinoma cell line, MDA-MB-231, and compared these phenotypes with the parental cell population. We observed differences in gene expression among the different clones and were able to link many of these changes to alterations in cytokine-mediated intercellular signaling pathways. The importance of these alterations at the cellular level was demonstrated by clone-specific phenotypes in vitro and the metastatic potential in vivo. Based on these results, we propose a model in which clone-specific secretion and reception of factors allow synergistic growth and ultimately contribute to tumor progression and metastasis.

## Methods

### Cell culture and reagents

The study was conducted using the MDA-MB-231 cell line. This is an epithelial human breast cancer cell line, established from a pleural effusion of a 51-year-old Caucasian woman with metastatic breast adenocarcinoma. The MDA-MB-231 breast cancer cell line was purchased from the American Type Culture Collection (ATCC) and authenticated by DNA profiling using short tandem repeat (STR) (GenePrint® 10 System, Promega) at Genomics Core Facility, Instituto de Investigaciones Biomédicas “Alberto Sols” CSIC-UAM. Viral, bacterial and parasitic pathogen analysis by RT-PCR/PCR was performed at Dynamimed Research Company: no genetic material was detected. Cells were maintained at 37 °C in a 5% CO_2_ humidified incubator (AutoFlow UN-5510, Nuaire), in Dulbecco’s modified Eagle’s medium (DMEM) (Life Technologies) supplemented with 10% heat-inactivated fetal bovine serum (FBS) (Biowest) and antibiotics (penicillin, streptomycin) (Gibco®-Invitrogen). Cells were trypsinized/passaged every 2–3 days using TrypLE reagent (ThermoFisher Scientific). Cells were automatically counted using Countess cell counting chamber slides (Invitrogen) and an Eve Automatic cell counter (NanoEntek), excluding dead cells by trypan blue (Invitrogen) staining.

### Color-coding (transfection), clone isolation and fluorescence confirmation

Ubc-StarTrack plasmids and the vector containing the hyperactive transposase of the PiggyBac system (hyPBase) were kindly provided by Dr. López-Mascaraque. Ubc-StarTrack plasmids were generated as previously described [35]. MDA-MB-231 cells were treated with 20 μM chloroquine and transfected with 3.5 μg total UbC-StarTrack plasmid DNA plus 1.2 μg total HypBase (transposase) DNA, diluted in HBS (HEPES buffered saline). CaCl_2_ was added to the medium to a final concentration of 150 mM. The medium was changed after six hours of incubation.

After transfection, the Ubc-StarTrack plasmids and the transposase under the control of the ubiquitous CMV promoter enter the nucleus. The Ubc-StarTrack plasmids are flanked by two terminal repeat sequences (ITRs) that are recognized and cut by the HypBase. The released sequence is integrated into the genome of the transfected cell at TTAA repeat regions, allowing the cell to stably produce the fluorescent 161 protein (Additional file 1: Figure S1A). This system allows tracking by color-coding of a complete population derived from the transfected cell.

Forty-eight hours after transfection, cells were trypsinized and isolated by fluorescence activated cell sorting (High Speed Cell Sorter FacsAria (Becton Dickinson)). We isolated cell clones expressing mT-Sapphire, EGFP, and monomeric Kusabira Orange (mKO) integrable plasmids. Cells were individually seeded in a 96 well plate to obtain clonal cell lines. Non-fluorescent cells were recovered to be used as a control (MDA-MB-231). The transfection efficiency was lower than 1%, showing that skewing of the parental cell line population was avoided. The clonal cell lines GFP C3, mKO E10, and Sapphire D7 and the MDA-MB-231 parental cell line were reauthenticated as described before. Fluorescence was confirmed on confocal microscopy (Spectral Confocal Microscope FV1000 (Olympus) and FV10-ASW 4.2 software. For every fluorescent protein the following excitation and emission wavelength settings were used: Ex.405-Em.520 (Sapphire), Ex.488-Em.520 (GFP), and Ex.515-Em.527 (mKO). After every trypsinization the percentage of fluorescent cells was determinate by flow cytometry (LSR Fortessa (BD), software FCS Express DeNovo), ruling out the possibility of fluorescent gene silencing.

### Contrast phase image capture

Forty-eight hours after seeding, phase contrast images were taken using an Olympus FSX100 microscope (amplification 40X) and FSX-BSW software.

### Cell count

Proliferation was evaluated by direct cell count. Fifty thousand cells were seeded in a six-well plate. Cells were trypsinized and automatically counted at the following time-points: 0 (24 h after seeding), 24, 48, and 72 h.

To calculate the cumulative population doublings (CPDs), 500,000 cells were seeded in a 100x17mm dish and trypsinized after 7 days in culture. Cells were counted and seeded again, repeating the same process for 4 weeks (28 days in culture). Cumulative population doublings were calculated using the following formula:

CPDs = [log(n° cells after 7 days) – log(n° seeded cells)] / log10^2^.

### Metabolic activity quantification (MTT assay)

Metabolic activity was evaluated by the MTT (3-[4,5-dimethylthiazol-25-yl]-2.5-diphenyl tetrazolium bromide; Panreac-AppliChem) assay. 2500 cells in 150 μL were seeded in 96-well plates in triplicate and the MTT assay was performed at time-point 0 (24 h after seeding), and at 24, 48 and 72 h. MTT was added to the medium to a final concentration of 0.5 mg/mL and incubated for 3 h at 37 °C. The medium was aspirated, and the formazan crystals were dissolved in 0.2 mL DMSO. Absorbance was measured at 595 nm using an Epoch Microplate Spectrophotometer (BioTek) and Gen 5 1.10 software.

### Spheroid formation assay

3D proliferation was evaluated by sphere growth measurements at 1, 4 and 7 days after seeding. Ten thousand cells per well were grown in ultra-low attachment 96 well plates in the presence of 5% Matrigel® (v/v) after 10 min of centrifugation at 2000 rpm. Pictures were taken with an inverted microscope NIKON Eclipse TE2000–5 and processed by ImageJ software to measure volume growth.

### Caspase assay

Caspase activity was measured using the Caspase-Family Colorimetric Substrate Set (K132, Biovision). Five hundred thousand cells were seeded in a 100x17mm dish and trypsinized after 96 h in culture. The pellet was resuspended in Cell Lysis Buffer (Biovision) and protein was quantified by Pierce™ BCA Protein Assay Kit (23,225, Thermo Fisher). A positive control was included: MDA-MB-231 cell line treated with Camptothecin (208,925, Merck) to a final concentration of 25 μg/mL for 24 h. 100 μg protein and *p*NA conjugated substrate to a final concentration of 0.5 mg/mL were added to each well in triplicate and incubated overnight at 37 °C. Absorbance was measured at 405 nm with an Epoch Microplate Spectrophotometer (BioTek).

### Spheroid invasion assay

3D invasion assay was performed by embedding 4-day-old spheres of each clone in a Matrigel-Collagen I mixture on a pre-coated 24-well plate with the same mixture. Sphere invasion was analyzed by measuring the invaded area at 24 and 48 h using the inverted microscope NIKON Eclipse TE2000–5 and ImageJ software.

### Migration assay

Five hundred thousand cells were seeded per well in 24-well plates in triplicate. After 24 h, the medium was replaced by medium without FBS and maintained overnight. Then, a wound was made in the monolayer with a pipette tip, and the medium was replaced with complete medium. Pictures of the wounds were taken at 0 h and 8 h using an Olympus FSX100 microscope. Wound closure was measured using ImageJ software. Results represent the migrated distance between 0 and 8 h, expressed as a percentage relative to MDA-MB-231.

### Transposon insertion event detection and characterization

Paired-end sequencing raw reads were first processed to remove PCR duplicates using Fastuniq. Then, we trimmed the specific Inverted Repeat (IR) associated with the transposon insertion process (GATTATCTTTCTAGGGTTAA, length = 20) with Trimmomatic [36]. This step was required to later select those reads that were shorter than the original read length (151), implying that they were covering a transposition event. To map the reads, we aligned the reads to the human reference genome g1k_v37 [37] using Bowtie 2 and allowing 1 mismatch [38]. Later, we filtered the alignment with SAMtools [39] to select paired reads mapped unambiguously with a minimum alignment quality of 30. The final step of the process relied on basic shell text processing tools (awk/grep/sed) to subset those pairs where one of the reads presented a length ≤ 131 (expected read length after removing the IR) and extract them from a specific position in the chromosome representing the first mapped genome base contiguous to the IR. This list of positions required an additional prioritization step as the IR sequence was endogenously found in the human genome. To differentiate these artefactual cases from genuine transposition events we took advantage of one of the transposase properties: they do not perform even cuts in both reverse/forward strands but a staggered cut that generates a duplication of 5 bases. A consequence of this is the representation of a specific insertion event at two positions: n and n + 5. This criterion was used to depict actual transposition events and define the set of insertion events that passed to the last step of characterization. In this last process, we used BEDTools map function and the annotation related to the human genome reference selected to map the specific insertion positions to their genomic context [40].

### Analysis of the transcribed genome

The microarray service was carried out at the High Technology Unit (UAT) at Vall d’Hebron Research Institute (VHIR), Barcelona (Spain). Affymetrix GeneTitan microarray platform and the Genechip Human Clariom D array cartridges were used for this experiment. This array analyzes gene expression patterns on a whole-genome scale on a single array with probes covering many exons on the target genomes, and thus permits an accurate summary of gene expression.

1.5 × 10^6^ cells were seeded in a 100x17mm dish in triplicate. After 72 h cells were trypsinized and centrifuged. RNA was extracted using the PureLink™ RNA Mini Kit (ThermoFisher). Quantification and assessment of RNA purity was performed using a NanoDrop™ 2000/2000c Spectrophotometer and confirmed according to the RIN (RNA Integrity Number) using RNA 6000 Nano Kit and 2100 Bioanalyzer (Agilent). Starting material was 200 ng of total RNA of each sample. Briefly, sense ssDNA suitable for labelling was generated from total RNA with the GeneChip WT Plus Reagent Kit from Affymetrix (Thermofisher-Affymetrix) according to the manufacturer’s instructions. Sense ssDNA was fragmented, labelled and hybridized to the arrays with the GeneChip WT Plus Terminal Labeling and Hybridization Kit from the same manufacturer.

All microarray data in this publication have been deposited in the NCBI Gene Expression Omnibus and are accessible through the GEO Series accession number GSE122008 (https://www.ncbi.nlm.nih.gov/geo/query/acc.cgi?acc=GSE122008).

Bioinformatic analysis was performed at the Statistics and Bioinformatics Unit (UEB) of the Vall d’Hebron Research Institute (VHIR, Barcelona, Spain). A Robust Multi-array Average (RMA) algorithm [41] was used for pre-processing microarray data. Background adjustment, normalization and summarization of raw core probe expression values were defined so that the exon level values were averaged to yield one expression value per gene. Data were subjected to non-specific filtering to remove low variability genes. Conservative thresholds were used to reduce possible false negative results. Selection of differentially expressed genes was based on a linear model analysis with empirical Bayes modification for the variance estimates [42]. To account for multiple testing, *P*-values were adjusted to obtain stronger control over the false discovery rate (FDR), as described by the Benjamini and Hochberg method. The analysis of biological significance was based on enrichment analysis against the Gene Ontology (GO; http://www.geneontology.org) and KEGG (http://www.genome.ad.jp/kegg/) databases.

### DNA methylation analysis

Microarray-based DNA methylation analysis was conducted with the Infinium MethylationEPIC BeadChip microarray (Illumina, San Diego, CA), that covers over 850,000 CpG methylation sites (850 K). 1.5 × 10^6^ cells were seeded in a 100x17mm dish in triplicate. After 72 h cells were trypsinized and centrifuged. Pellets were frozen until DNA extraction. DNA quality checks, bisulfite modification, hybridization, data normalization, statistical filtering, and beta (β) value calculations were performed as described elsewhere [43, 44]. The DNA concentration of the samples was measured using the Quantifluor ONE dsDNA System (Promega). A total of 500 ng of DNA samples were selected for bisulfite conversion with the EZ DNA Methylation™ Kit (Zymo Research). The Illumina Infinium HD methylation protocol was followed for the hybridization to the Infinium MethylationEPIC BeadChips. Whole-genome amplification and hybridization were performed on the BeadChips and followed by single-base extension and analysis on a HiScan (Illumina, San Diego, CA) to assess the cytosine methylation states. The methylation score of each CpG was represented as β value, and previously normalized for color bias adjustment, background level adjustment and quantile normalization across arrays. Probes and sample filtering involved a two-step process for removing SNPs and unreliable betas with a high detection *P*-value > 0.01. Sex chromosome probes were also removed. After this filtering, the remaining CpGs were considered valid for the study.

### Cytokine quantification

1.5 × 10^6^ cells were seeded in a 100x17mm dish in triplicate. After 72 h the medium was collected and filtered through 0.22 μm filters (Merck Millipore) to remove floating cells, apoptotic bodies and cell debris. The concentrations of the cytokines TNF alpha, IFN gamma, IL-1 beta, IL-2, IL-4, IL-5, IL-6, IL-8, IL-9, IL-10, IL-12p70, IL-13, IL-17A, GM-CSF, MCP1, MIP1a and MIP1b were determined using the FirePlex Human Key Cytokines - Immunoassay Panel (ab229791) by Abcam FirePlex Service Lab.

### Complementation assays

To test the presence of factors that could increase proliferation and metabolic activity, the media where the parental cell line or a clone were cultured for several days were recovered. The medium was tested by two independent assays (MTT and cell count). Exosomes were tested by MTT. “Donors cells” were cultured as follows: 17,600 cells/cm^2^ (medium complementation) or 10,400 cells/cm^2^ (exosome complementation) were seeded in 0.17 or 0.1 mL complete medium/cm^2^, respectively. Twenty-four hours before treatment the “receptor cells” were seeded: 1250 cells in 150 μL were seeded in 96-well plates in triplicate (MTT assay) or 25,000 cells in 3 mL were seeded in 6-well plates in duplicate (cell count). Seventy-two hours after the “donor” seeding, the medium was recovered. The medium was filtered through 0.22 μm filters (Merck Millipore) to remove possible floating cells and added to the “receptor cells” (complete medium, CM). The medium was also ultracentrifuged to extract exosomes, the supernatant after two ultracentrifugations was recovered (soluble factors, SF) and the pellets were resuspended in PBS (exosomes). Exosome protein concentration was quantified by BCA Protein Assay kit (ThermoFisher).

To perform the direct cell count, donor medium was added to new medium to a final concentration of 0, 10, 25 and 50%. Cells were counted after 96 h’ treatment.

For the MTT assay, donor medium was added to new medium to a final concentration of 0, 25 and 50%. MTT assay was performed at time-point 0 (24 h after seeding), 72 and 96 h. To study the effect of exosomes on cell metabolic activity, cells were treated with 50% complete medium and 50% soluble factors as a control. Exosomes were added to a final concentration of 5, 10, 20, 40, 80 and 160 μg protein/mL. MTT assay was performed at 72 h.

### Exosome isolation

Seventeen thousand six hundred cells/cm^2^ were seeded in 0.1 mL complete medium/cm^2^. After 72 h in culture, supernatant was recovered and sequentially centrifuged as follows: 500 rcf/10 min, 12,000 rcf/20 min and 100,000 rcf/90 min (× 2). Finally, each pellet was resuspended in PBS.

### Protein extraction and immunoblotting

Total protein extracts were generated using RIPA Lysis Buffer System (sc24948, SantaCruz Biotechnology) supplemented with Protease Inhibitor Cocktail set III (539134) and Phosphatase Inhibitor Cocktail Set II (524625) from Calbiochem. Protein was quantified using a BCA Protein Assay kit (23,225, ThermoFisher). Protein extracts (10 μg per sample) were loaded onto SDS-PAGE gels and electrophoretically transferred to PVDF membranes. The following primary antibodies were used: CD81 (sc-166,028, SantaCruz Biotechnology), TSG101 (ab83, Abcam) and b-actin (JLA20, Calbiochem). Goat anti-rabbit and anti-mouse HRP secondary antibodies were from Pierce ThermoScientific (31460) and Calbiochem (JA1200), respectively. Immunodetection of proteins was performed using Amersham ECL Western Blotting Detection Reagent (GE Healthcare).

### Transmission electron microscopy

Exosomes were characterized by transmission electron microscopy (TEM), using negative staining. Electron microscopy images were recorded on a T20-FEI Tecnai thermoionic microscope operated at an acceleration voltage of 200 kV. Negative stained samples were prepared by dropping 20 μL of sample onto carbon coated copper grids (200 mesh), which were then dried at room temperature and stained with phosphotungstic acid. Exosome size was measured using ImageJ software.

### Co-culture assay

MDA-MB-231, GFP C3, mKO E10 and Sapphire D7 cell lines were cultured as individual cell lines and as combinations, with 2, 3 and 4 cell lines in co-culture. The co-cultures started with equal percentages of every clone. Five hundred thousand total cells were seeded in a 100x17mm dish and trypsinized after 7 days in culture. Cells were counted and seeded again, repeating the same process for 4 weeks (28 days in culture). After every trypsinization the percentage of every cell line per co-culture was determinate by flow cytometry (LSR Fortessa (BD), software FCS Express DeNovo). CPDs were calculated as previously described. Expected CPDs were calculated using data from the individual plates (single culture). Observed CPDs were calculated using data from every co-culture.

### Arsenite resistance assay

Two hundred thousand total cells were seeded in a 60x17mm dish. 24 h after seeding, cells were treated for 90 min with 250 μM Arsenite (NaAsO2 in complete medium). After treatment, cells were washed twice with PBS and new complete medium was added. Cells were counted after 72 h.

The co-culture experiment was performed in the presence of arsenite. The co-cultures started with equal percentages of every clone (100,000 cells per clone): MDA-MB-231 + GFP C3 or MDA-MB-231 + mKO E10. 24 h after seeding, cells were treated as detailed before. The percentage of each cell line in the total population was detected by flow cytometry at seeding (day 0), 90 min (day 1) and 72 h (day 4) after treatment.

### Invasion assay

8-μm pore inserts were covered with 65 μL Matrigel (356,231, Corning) at a final concentration of 1.5 mg/mL. Matrigel was incubated for 4 h at 37 °C. Once the Matrigel was polymerized over the Matrigel layer, 30,000 cells were seeded in 100 μL medium without FBS. Complete medium (supplemented with FBS, which stimulates the cells to cross the Matrigel layer) was added to the well under the insert, covering the bottom of the insert. Twenty-four hours after seeding, cells were fixed with PFA 4% and counterstained with Hoechst. The top of the insert was cleaned with a cotton bud to remove the Matrigel and any cells that did not cross the layer. Images were taken of the entire insert bottom where the invading cells were located, using an Olympus FSX100 microscope (amplification 4.2X) and FSX-BSW software.

Following the previous description, we performed two different invasion assays, with co-culture and with medium complementation. Modifications to the previous protocol are detailed as follows:

1. Co-culture: GFP C3 and mKO E10 were seeded as single cell lines and as an equal combination of both cell lines.
2. Medium complementation: GFP C3 and mKO E10 were seeded as single cell lines. Complemented complete medium was added to the well under the insert, covering the bottom of the insert. Complemented medium was obtained as detailed in “Complementation assays”.

### Animal experimentation

Female athymic nude mice (Strain: Hsd:Athymic Nude-Foxn1nu) (ENVIGO, Spain) were kept in pathogen-free conditions and used at 7 weeks of age. Animals were randomly housed under Specific Pathogen Free (SPF) conditions in autoventilated racks in groups of six. Two enrichment elements were included, a cardboard tube and a square nestlet for nesting and thermoregulation. The housing temperature ranged from 23 °C to 25 °C, relative humidity ranged between 47 and 55%. The cycle gradually simulates twilight and sunset, giving 12 h of light with an intensity of 300 Lux and 12 h of darkness for each 24 h period. Food and water were provided ad-libitum. Animal care was handled in accordance with the Guide for the Care and Use of Laboratory Animals of the Vall d’Hebron University Hospital Animal Facility, and the experimental procedures were approved by the Animal Experimentation Ethics Committee at the institution (76/17 CEEA). Body weight and physical appearance of the animals were monitored twice a week. The animals were euthanized by cervical dislocation following the euthanasia standard operating procedure (SOP) of Laboratory Animals of the Vall d’Hebron University Hospital Animal Facility. All the in vivo studies were performed by the ICTS ‘NANBIOSIS’, more specifically, by the CIBER-BBN in vivo Experimental Platform of the Functional Validation & Preclinical Research (FVPR) area (Barcelona, Spain).

The implanted cells were the MDA-MB-231 parental cell line, three in vitro isolated clones (mKO E10, GFP C3 and Sapphire D7), both individually and as a mix of the four lines in a ¼ ratio. The parental cell line was included in the mix to provide factors probably not produced by the clonal cell lines and which may be needed for tumor growth and/or metastasis. All cell variants were confirmed to be negative for viral, bacterial (including mycoplasma) and parasitic pathogens (VHIR Screening Humano Completo). The test was performed by the external reference laboratory Dynamimed Research Company. Prior to the injection, the percentage of fluorescent cells was quantified by flow cytometry, to confirm the injected cells expressed the fluorophore and exclude the possibility of fluorescent gene silencing.

The tumor growth rate and invasive capacities were evaluated by implantation of 2.5 × 10^6^ cells into the right abdominal mammary fat pad (i.m.p.f.) of twelve animals per group (five groups). The tumor volume was measured by caliper measurements twice a week and calculated according to the formula *D × d2/2*, where *D* is the largest diameter of the tumor and *d* the smallest one. All animals were euthanized 34 days after inoculation to compare the primary tumor size and composition and the number and extent of lung metastases between groups. The tumors and lungs were weighed, fixed with paraformaldehyde 4%, and later processed for histopathological analyses (hematoxylin and eosin staining).

The metastasis growth rate of the MDA-MB-231 and clonal cell lines was evaluated by intravenous (IV) injection of 2.5 × 10^6^ cells into the caudal tail vein of 10 animals per group (five groups). All animals were euthanized 36 days after inoculation. Animals underwent gross necropsy consisting of a macroscopic evaluation. Lungs were excised, weighed, fixed and processed for histopathological analysis.

Immediately following dissection, the tumors and lungs were fixed for 24 h by immersion in paraformaldehyde 4%. After fixation, the tissue was dehydrated to enable embedding with paraffin. Five-micron-thick sections were cut from fixed, paraffin-embedded tissues and mounted on poly-L-lysine-coated glass slides. Sections were deparaffinized in xylene and rehydrated in graded alcohol. The presence of clonal cells in the primary tumor and metastases were detected by fluorescence on confocal microscopy (Spectral Confocal Microscope FV1000 (Olympus)). To avoid spectral overlapping of the different fluorescent proteins, a Lambda scan was performed from 470 to 635 nm followed by spectral deconvolution using FV10-ASW 4.2 software. Images were quantified using the program ImageJ.

### In vivo zebrafish tumor xenograft assays

Zebrafish (*Danio rerio*) embryos were generated by natural mating of adult fish according to previously described procedures (Westerfield, 2000). In order to remove surface pigmentation, embryos were incubated with PTU (N-Phenylthiourea, Sigma) at a 0.003% w/v concentration and maintained at 28 °C. For xenograft experiments, 2 days postfertilization (dpf) zebrafish embryos were de-chorionized if necessary and anaesthetized with 0.003% tricaine (Sigma) w/v in E3 water and positioned on a 10 cm Petri dish coated with 1.5% agarose. Immediately prior to injection, single cell suspensions of MDA-MB-231-GFP C3 cells and MDA-MB-231-mKO E10 cells were labelled with the lipophilic fluorescent tracking dyes CM-DiD and CM-DiI (Invitrogen) respectively, according to manufacturer’s instructions. To remove unincorporated dye, cells were centrifuged and rinsed twice with Dulbecco’s phosphate-buffered saline (DPBS). Cells were kept on ice before implantation and implanted within 3 h. For microinjection, the individual cell populations (individual injection) or the combination of both at equal numbers (co-injection) were loaded in borosilicate glass capillary needles (1 mm O.D. × 0.58 mm I.D, Harvard Apparatus) and approximately 300 cells were injected into the duct of Cuvier (DoC) using a micromanipulator and an IM 300 microinjector (Narishige) with an output pressure of 10 psi and 0.03 ms injection time. After injection, embryos were examined for the presence of a fluorescent cell mass at the injection site in the DoC, and then transferred to fresh PTU-containing E3 water and placed into an incubator at 34 °C for up to 3 days post injection (dpi). Zebrafish embryos were photographed at 0 h post injection (hpi) and 72 hpi with a fluorescent microscope DMi8 (Leica) to determine tumor cell dissemination. The number of tumor cells disseminated in the tail vein of the fish was analyzed with the software ImageJ Fiji. For each condition, data are representative of at least three independent experiments, with at least 30 embryos per group.

### Statistics

Prism 5.0 software (GraphPad Software Inc., La Jolla, CA) was used for statistics and data representation. Significant differences were determined using unpaired t-test with Welch’s correction (equal SDs not assumed) and ANOVA (Tukey’s multiple comparisons test). Individual figure legends specify the test used in each case. Asterisks indicate significant differences when *P*-values are < 0.05 (*), < 0.01 (**), and < 0.001 (***).

### Graphics

Graphics were created using Prism 5.0 software and Adobe® Illustrator CC.

## Results

### Generation of color-coded clonal cell lines derived from the MDA-MB-231 cell line

In order to study tumor heterogeneity in a cell culture-based model, we selected the triple negative human breast cancer (TNBC) cell line MDA-MB-231. The presence of genetically and phenotypically different clonal populations has previously been described in this line [45]. To allow us to study individual clonal populations and trace their fate during tumor progression, cells were color-coded according to the expression of fluorescent proteins [46, 47]. Genomic integration of fluorescent protein-encoding sequences was achieved by the use of the UbC-StarTrack PiggyBac transposon system (Additional file 1: Figure S1a) [35, 46]. To generate the color-coded cell lines, we used different transposon-containing plasmids that encode for the fluorescent proteins mT-Sapphire, EGFP and mKO (Additional file 1: Figure S1b). From the clonal cell lines, we selected GFP C3 (EGFP), mKO E10 (mKO) and Sapphire D7 (mT-Sapphire) for further study, each of which expresses one fluorescent protein (Additional file 1: Figure S1c). Additionally, FACS-sorted non-fluorescent cells were pooled and grown as a control population (named MDA-MB-231, parental). The transfection efficiency was lower than 1%, showing that skewing of the parental cell line composition was avoided. Finally, cell line authentication by DNA profiling of short tandem repeats (STR) confirmed that all obtained clones were derived from the parental cell line MDA-MB-231 (Table 1).

**Table 1:** Authentication profile for the selected clones vs. MDA-MB-231 parental cell line. Characterization of the clonal cell lines (GFP C3, mKO E10 and Sapphire D7) versus parental cell line (MDA-MB-231, also named ATCC® HTB-26) by STR profile evaluation (first column): number of shared alleles and percent match

STR profile	Expected: ATCC® HTB-26	MDA-MB-231	GFP C3	mKO E10	Sapphire D7
D5S818	12	12	12	12	12
D13S317	13	13	13	13	13
D7S820	8,9	8	8	8	8
D16S539	12	12	12	12	12
vWA	15,18	15	15	15,16	15
TH01	7,9.3	7,9.3	7,9.3	7,9.3	7,9.3
TPOX	8,9	8,9	8,9	8,9	8,9
CSF1PO	12,13	12,13	12,13	12,13	12,13
D21S11	30,33.2	30,33.2	30,33.2	30,33.2	30,33.2
Amelogenin	x	x	x	x	x
Number of shared alleles		14	14	14	14
Number of alleles in database		16	16	16	16
Percent match		87%	87%	87%	87%

To confirm the presence of heterogeneity in the MDA-MB-231 cell line and to validate our approach of studying tumor heterogeneity in a cell line-based model, we characterized different phenotypic features of the individual clonal cell lines**.**

#### Cell morphology

**Fig. 1:**
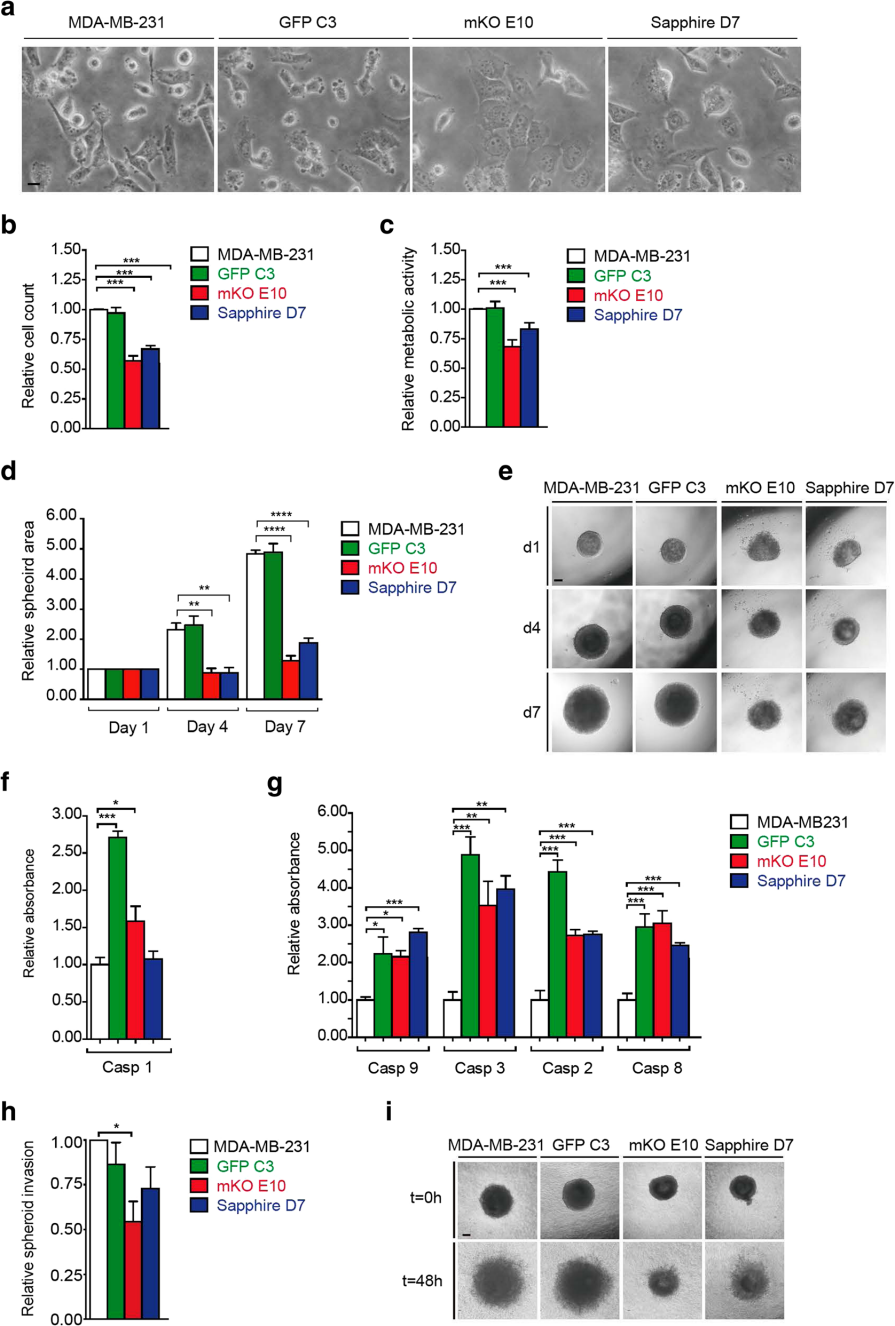
Phenotypic characterization of MDA-MB-231, GFP C3, mKO E10, and Sapphire D7 cell lines. **a** Morphological evaluation by phase contrast images (scale bar = 200 μm). **b** Cell count after 72 h in culture, relative to MDA-MB-231 count. **c** Metabolic activity rate: quantification of MTT metabolization by metabolically active cells, absorbance relative to MDA-MB-231. **d-e** 3D growth rate: spheroid area at days 1, 4 and 7, relative to MDA-MB-231-day 0 quantification (d) and representative pictures (scale bar = 250 μm) (e). **f-g** Apoptotic analysis: absorbance relative to MDA-MB231. Expression of Caspase 1 (f) and Caspase 9, 3, 2 and 8 (g). **h-i** 3D invasion by spheroid cell migration: relative invasion (h) and representative picture at 0 and 48 h (scale bar = 250 μm) (i). Significant differences were determined using unpaired t-test with Welch’s correction. Asterisks indicate significant differences when *P*-values are < 0.05 (*), < 0.01 (**), and < 0.001 (***)

MDA-MB-231 is an epithelial cell line, characterized by elongated polygonal cells. Although all clonal cell lines maintained an epithelial morphology, the shape of the cells varied between the different clones, the clone mKO E10 being the most different to the others, with more polygonal and less elongated morphology (Fig. 1a, Additional file 2: Figure S2a).

#### Cell growth

Cell growth was determined by counting the number of viable cells in an exponentially growing culture 72 h after seeding. As shown in Fig. 1b, the growth rate of GFP C3 was similar to that of the parental cell line. In contrast, the clones mKO E10 and Sapphire D7 had a reduced growth rate of up to 40 and 30%, respectively, compared to the parental cell line. These results were confirmed by measuring the metabolic activity of the cells via MTT assay (Fig. 1c). To confirm these results were not a consequence of 2D culture, we performed spheroid 3D culture. The results were confirmed, showing an even greater growth rate reduction in 3D culture than in 2D culture for mKO E10 and Sapphire D7 (Fig. 1d-e). With the aim of determining if this phenotype is heritable, we cultured the different cell lines for 28 days and calculated the cumulative populations doublings (CPDs) after 7, 14, 21 and 28 days in culture (Additional file 2: Figure S2b). Cell lines maintained stable proliferation rates over time.

#### Apoptosis

In order to assess if the reduction in cell growth was due to increased cell death, we determined the activation of caspase 1 activity, known to induce pyroptosis, a lytic form of cell death. While the clone mKO E10 showed some activation of caspase 1, a striking increase of caspase 1 activity was observed in the clone GFP-C3 (Fig. 1f). The activity of caspases [2, 3, 6, 9] was measured as a readout for the activation of pro-apoptotic signaling pathways. In comparison to the parental cell line MB-MDA231, cells of the clonal cell lines GFP C3, mKO E10 and Sapphire D7 exhibited significantly higher levels of these caspases (Fig. 1g).

Cell growth was measured by cell counting after 72 h in culture. Cell growth is the net result of proliferation and cell death. GFP C3 may have had increased apoptosis that was compensated by higher proliferation; in contrast, cell growth in low-growth-rate cell lines would be the result of decreased proliferation, rather than increased apoptosis.

#### Cell migration

The wound healing assay was used to measure cell migration, which is essential for many biological processes including tumor invasion and metastasis [48]. The analyzed clonal cell lines had similar migratory capacity to the parental cell line (Additional file 2: Figure S2c-d).

#### Invasion

Spheres assembled in 3D cultures were embedded in a Matrigel/collagen matrix and the expansion of the spheres into the matrix was determined microscopically. Analysis of the obtained data revealed that, while the invasive capacity of the clones GFP and Sapphire was similar to the parental cell line, cells from the clone mKO had a reduced invasion rate of up to 50% into the matrix (Fig. 1h-i).

### Tumorigenicity and metastasis of clonal cell lines

#### Tumorigenicity of clonal cell lines

**Fig. 2:**
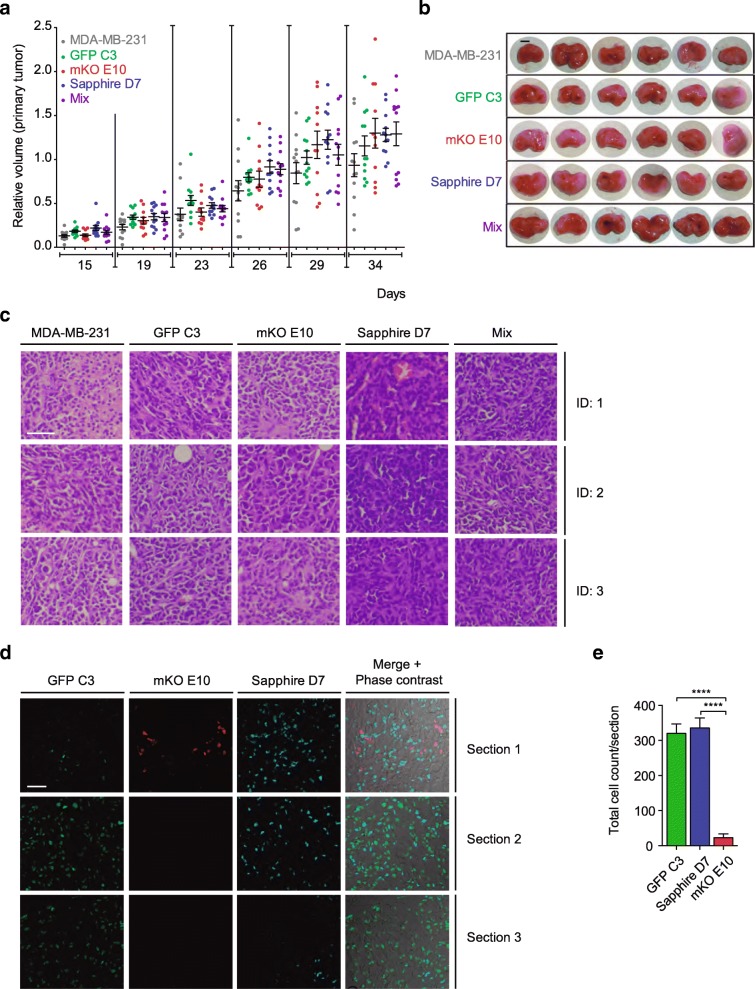
Malignancy of individual clones and tumor heterogeneity. **a-c** Tumor growth over 34 days after orthotopic injection of MDA-MB-231, GFP C3, mKO E10, or Sapphire D7 clonal cell lines and an equal mix of the previous cell lines (mix): (a) volume relative to MDA-MB-231 tumors (*n* = 12 per group), (b) macroscopic images of the tumors (scale bar = 0.5 cm) and (c) hematoxylin/eosin stains of tumor sections (scale bar = 50 μm) (three different animals per cell line: ID1, ID2, ID3). **d-e** Tumor heterogeneity: (d) representative fluorescent images (scale bar = 50 μm) of three independent sections of tumor formed by MDA-MB-231 parental and GFP C3, mKO E10 and Sapphire clonal cell lines (mix) and (e) quantification of tumor composition formed by the clonal cell lines (*n* = 6). Significant differences were determined using unpaired t-test with Welch’s correction. Asterisks indicate significant differences when *P*-values are < 0.05 (*), < 0.01 (**), and < 0.001 (***)

Having established that the isolated clonal cell lines displayed phenotypic heterogeneity in vitro, we next analyzed their tumorigenic potential in vivo. We orthotopically injected the clones individually (GFP C3, mKO E10, Sapphire D7 or the parental MDA-MB-231 cell line) and in combination (mix of clones and parental cell line, 25% each) into the mammary fat pad of immunocompromised nude mice. Surprisingly, despite the differences observed for these clones in vitro, all of the clonal cell lines formed primary tumors, and the tumor growth rates were similar for all individual clonal lines and the parental cell line (Fig. 2a-b). Similar results were observed when the cell clones and parental cell line were co-injected as a mixture (Fig. 2a-b).

Despite the similar tumor growth rates, we wondered if the tumors would have morphological differences. Pathological examination of the tumors revealed that the primary tumor cells from the different cells lines were of a similar shape, but with subtle differences. Cells within the mKO E10-derived tumors displayed a more rhabdoid, plasmacytoid shape, Sapphire D7 being the more compact tumor, formed by particularly small, round cells (Fig. 2c). Thus, despite similar tumor growth rates, tumor cell shape varied slightly, depending on the origin of the clones.

The tumors formed after co-injection of the different clonal cell lines and the parental cell line were assessed on confocal microscopy. As shown in Fig. 2d-e, the clonal composition varied depending on the section analyzed, and the individual clones showed variable intermixing (Fig. 2d). Figure 2e shows the number of fluorescent clonal cells and the parental cells (not expressing fluorescent proteins) in the analyzed sections. Interestingly, the parental cells were predominant, while the clones GFP C3 and Sapphire D7 were detected in various areas of the tumor and few mKO E10 cells were observed (Fig. 2d-e). Finally, cell counting of different sections of the whole tumor revealed that the majority of fluorescent clone cells within the tumor were either GFP or Sapphire positive (46.96 and 49.74%, respectively) and only 3.3% of the cells were derived from the clone mKO E10 (Fig. 2e). Considering that all the clones had similar growth rates when injected individually into the mice, this finding indicates that the interplay among the different clones determines their fate during tumor progression.

#### Metastatic tumor formation capacity

**Fig. 3:**
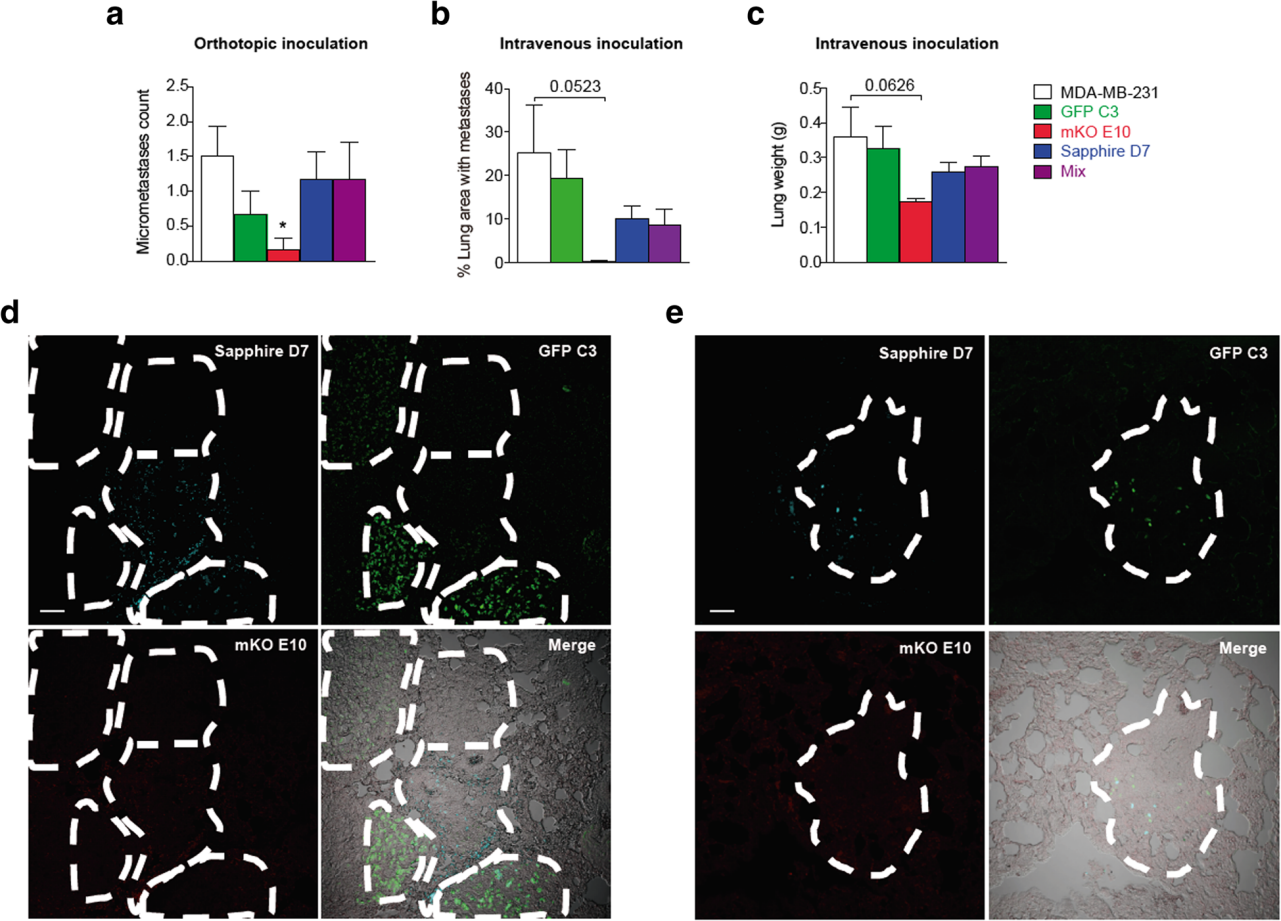
Metastatic capacity of cell lines and an equal mix of all cell lines. **a** Absolute count of micrometastases derived from orthotopic primary tumors, 34 days after injection of tumor cells. **b-c** Metastasis growth induced 35 days after intravenous injection of tumor cells: (b) lung area occupied by metastases, and (c) lung weight, an indirect measurement of lung tumors. Significant differences were determined using unpaired t-test with Welch’s correction. Asterisks indicate significant differences when P-values are < 0.05 (*), < 0.01 (**), and < 0.001 (***). **d-e** Representative fluorescent images (scale bar = 50 μm) of four independent sections of lung metastasis formed by MDA-MB-231 parental and GFP C3, mKO E10 and Sapphire clonal cell lines (mix)

We next analyzed the ability of the clones to form metastases derived from the orthotopic tumor. The lungs of the sacrificed mice were stained with hematoxylin/eosin, and microscopy revealed that, although cells from all clonal cell lines were able to form metastases, the metastasis count was significantly lower in the mKO E10 clonal cell line (Fig. 3a).

To confirm these results, we determined the metastatic capacity of the individual cell lines in the lungs of the mice after tail vein injection. Measurement of the metastatic areas within the lungs confirmed the reduced capacity of mKO E10 to form metastases (Fig. 3b, Additional file 3: Figure S3). The lungs were weighed as an indirect measurement of the presence of metastases, and the results were confirmed by area measurement (Fig. 3c). These results indicate that the inability of these cells to form metastases might be due to their homing or survival in the lungs. We think that the mKO E10 cell line would be an appropriate subject of future research on targeted therapies.

Hematoxylin/eosin stained lung sections confirmed inter-individual heterogeneity, in the percentage of lung area occupied by metastases formed by MDA-MB-231 (Mean ± SEM 25.14% ± 11.14), GFP C3, (19.42% ± 6.41), mKO E10 (0.25% ± 0.20), Sapphire D7 (10.07% ± 2.95) and Mix (8.54% ± 3.78) (Fig. 3b). Photographs showing the variability among individual animals are shown in Additional file 3: Figure S3.

In addition to determining the metastatic capacity of the individual clones, our experimental setup of the mixed clonal populations also allowed us to determine if macrometastases were formed from individual or multiple cells. We used confocal microscopy to analyze the metastases induced by an equal mix of the parental cell line and clonal cell lines (Fig. 3d). mKO E10 cells were not found in the resulting metastases. Fluorescence revealed the metastases could be formed by one (Fig. 3d) or more than one clone (Fig. 3e). However, even in metastases formed by more than one clone, we found a main component formed from a single clone.

### Molecular characterization of clonal cell lines by gene expression, methylomics and cytokine expression

#### Gene expression profiling by microarray analysis

**Fig. 4:**
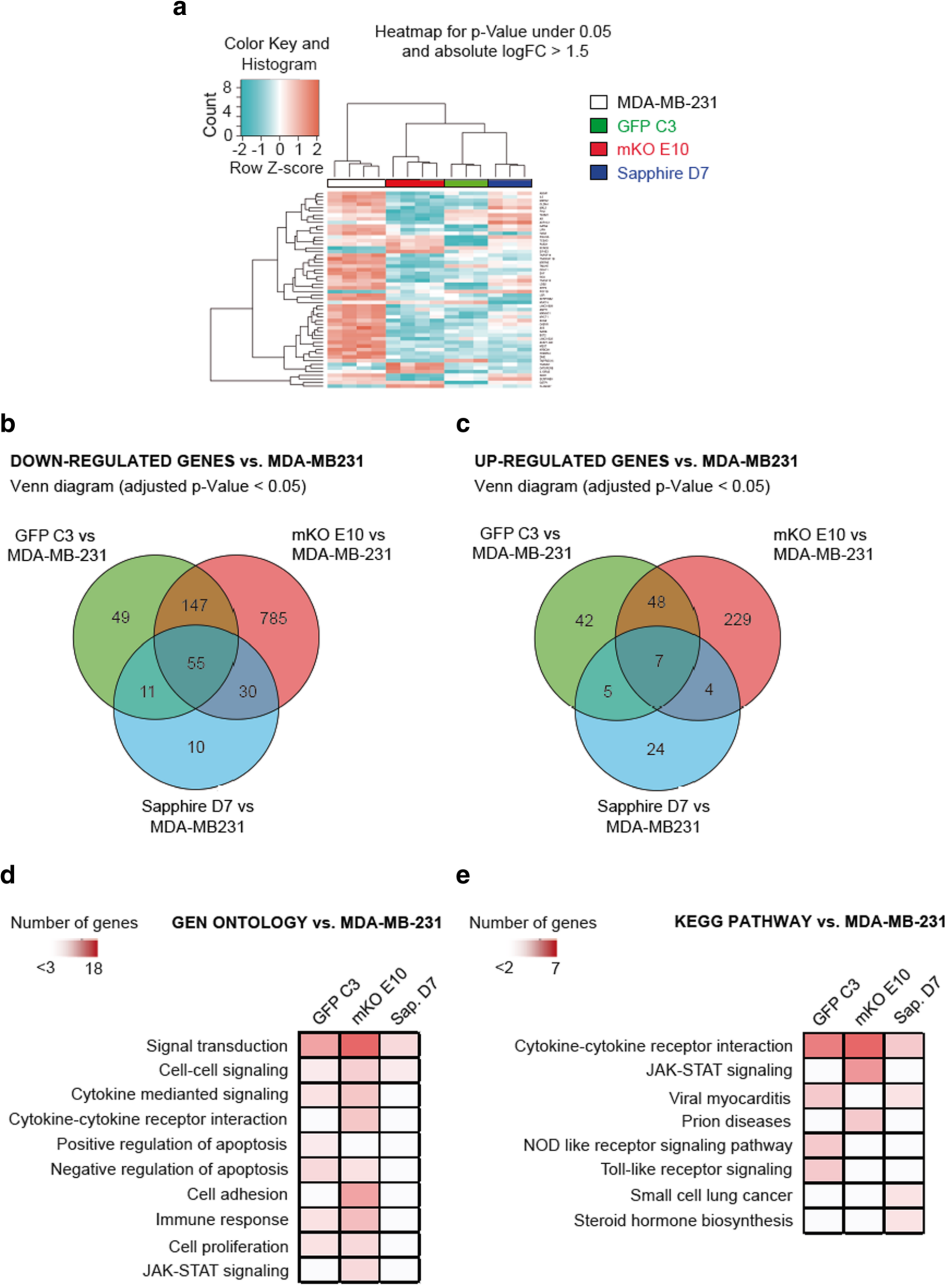
Transcriptional profile of MDA-MB-231 clonal cell lines. **a** Heatmap for P-value under 0.05 and absolute logFC> 1.5 of selected clonal cell lines. **b-c** Venn diagrams of downregulated (b) and upregulated (c) transcripts of selected clonal cell lines vs. MDA-MB-231. **d** Gene ontology (GO) with differentially expressed genes (logFC> 1 and FDR < 0.05) of each clonal cell line vs. MDA-MB-231 cell line. **e** KEGG with differentially expressed genes (logFC> 1 and FDR < 0.05) of each clonal cell line vs. MDA-MB-231 cell line

To uncover the molecular mechanisms of interplay between different clones in a heterogeneous population, microarray analysis was performed to determine the relative alterations in gene expression profiles of the clonal cell lines in comparison to the parental cell line MDA-MB-231(Fig. 4).

Variation at the level of individual transcripts was quantified by identifying which genes had a statistically significant difference in expression level from average (*p* < 0.1) and expressing these in a heatmap. As depicted in Fig. 4a, significant differences in the gene expression profiles were detected between the different clonal cell lines and the parental cell line. Statistically significant differences in gene expression compared to MDA-MB-231 cells were determined, separated into up- and downregulated genes and displayed as a Venn diagram (Fig. 4b-c). The most striking differences were observed for the clone mKO E10: 1017 genes were downregulated, and 288 genes were upregulated, while the clonal cell lines GFP C3 and Sapphire D7 had fewer regulated genes (GFP C3, 262 down and 71 up; Sapphire D7, 106 down and 40 up), in comparison to the parental cell line (Fig. 4b-c).

To elucidate which alterations in gene expression could potentially account for the observed phenotypic differences in vitro and in vivo, we performed bioinformatic analysis. We narrowed down the number of potential candidates using gene ontology and KEGG pathway analysis to identify the pathways and biological processes that were altered in the different clones (Fig. 4d-e). Gene ontology (GO) revealed both common and clone-specific changes in biological processes (Fig. 4d). Most of the changes occurred in the expression levels of genes involved in signaling cascades. Interestingly, many of the genes identified within the class of signaling factors also belonged to the GO term “cytokine-mediated signaling pathways”, the third most abundant class of pathway affected in the analysis. Therefore, intercellular communication via cytokines and their respective downstream signaling pathways displayed the greatest differences between clonal cell lines and the parental MDA-MB-231 cells. Following this line, we were able to identify cytokines and cytokine receptors that showed alterations that were either common to the parental cell line or clone-specific. The detection of specific signaling pathways in the analysis, namely NOD, Toll-like receptor and JAK-STAT signaling pathways, was also due to the cytokines and their receptors, acting upstream of the respective pathways. KEGG pathway analysis confirmed these findings (Fig. 4e).

To elucidate why the clone mKO E10 had a non-metastatic phenotype, we matched our dataset with the Human Cancer Metastasis Database. This analysis revealed that of the 658 genes with altered mRNA expression in the clone mKO, 95 have been linked to metastasis (Additional file 4: Table S1). Among the genes with the biggest alterations compared to the parental cell line was TGF-beta receptor type-1 (TGFR-1, log2FC -0.9). TGF-beta receptor signaling has been strongly linked to metastatic disease progression in breast cancer, so downregulation of TGFR-1 in the clone mKO might have been the reason for the non-metastatic phenotype [49, 50]. Detailed analysis of these and other potential factors will be required in the future to determine how their altered expression can affect metastatic disease progression.

#### Transposon sequencing

In order to test if the observed changes in gene expression profiles reflected a natural heterogeneity within the MDA-MB-231 cell line, or if these changes were induced by genomic alterations through the insertion of transposons, we performed genomic DNA sequencing, mapping the exact insertion sites of the transposons. We detected multiple insertions of the same transposon into the genomic DNA, but, excepting one insertion in the Sapphire D7 clone, these insertions were located in non-coding genomic regions (introns). Analysis of the microarray data confirmed none of the insertions affected the corresponding gene expression with respect to MDA-MB-231 parental cell line (Table 2).

**Table 2:** Location of transposons in the genome of MDA-MB-231 clonal cell lines. Chromosome location (chromosome number and position, region type, strand, gene name, intron/exon, number of isoforms with the insertion, analyzed or not in the microarray, microarray probe and Clone vs MDA-MB-231 logFC in microarray analysis

Cell line	Chromosome	posL	posR	Type	Strand	Name	Relative location	Isoforms	N° isoforms with the insertion	Microarray analysis	Microarray probe	Clone vs MDA-MB231 logFC
MDA-MB-231	–	–	–	–	–	–	–	–	–	–	–	–
GFP C3	10	33,272,491	33,272,496	Gene	Reverse	ITGB1	Intron	2	0	Yes	TC1000010265.hg.1	Non significant
				lncRNA	Forward	RP11-462 L8.1	Intron	2	0	No	–	
mKO E10	10	69,810,770	69,810,775	Gene	Reverse	HERC4	Intron	8	2	Yes	TC1000010831.hg.1	Non significant
	10	71,001,553	71,001,558	Gene	Forward	HKDC1	Intron	2	0	Yes	TC1000007890.hg.1	Non significant
	10	41,117,156	41,117,161	Gene	Reverse	PPP1R14D	Intron	2	0	Yes	TC1500009127.hg.1	Non significant
Sapphire D7	5	170,347,638	170,347,643	Gene	Forward	RANBP17	Intron	10	0	Yes	TC0500009411.hg.1	Non significant
	8	145,570,348	145,570,353	Intergenic	–	–	–	–	–	Yes	–	Non significant
	9	113,552,211	113,552,216	Gene	Forward	MUSK	Intron	3	0	Yes	TC0900008415.hg.1	Non significant
	9	78,576,637	78,576,642	Gene	Forward	PCSK5	Intron	3	0	Yes	TC0900007618.hg.1	Non significant
	10	112,724,387	112,724,392	Gene	Forward	SHOC2	Exon	5	3	Yes	TC1000008908.hg.1	Non significant
	X	106,242,426	106,242,431	Gene	Reverse	MORC4	Intron	3	0	Yes	TC0X00010459.hg.1	0.484

In summary, even though all clonal cell lines were derived from the same parental cell line, their mRNA expression profiles varied widely, and this variation was not caused by genomic mutations via transposon insertion. We can therefore conclude that heterogeneity within the MDA-MB-231 is present at the level of gene expression.

#### Epigenetic alterations of gene expression

**Fig. 5:**
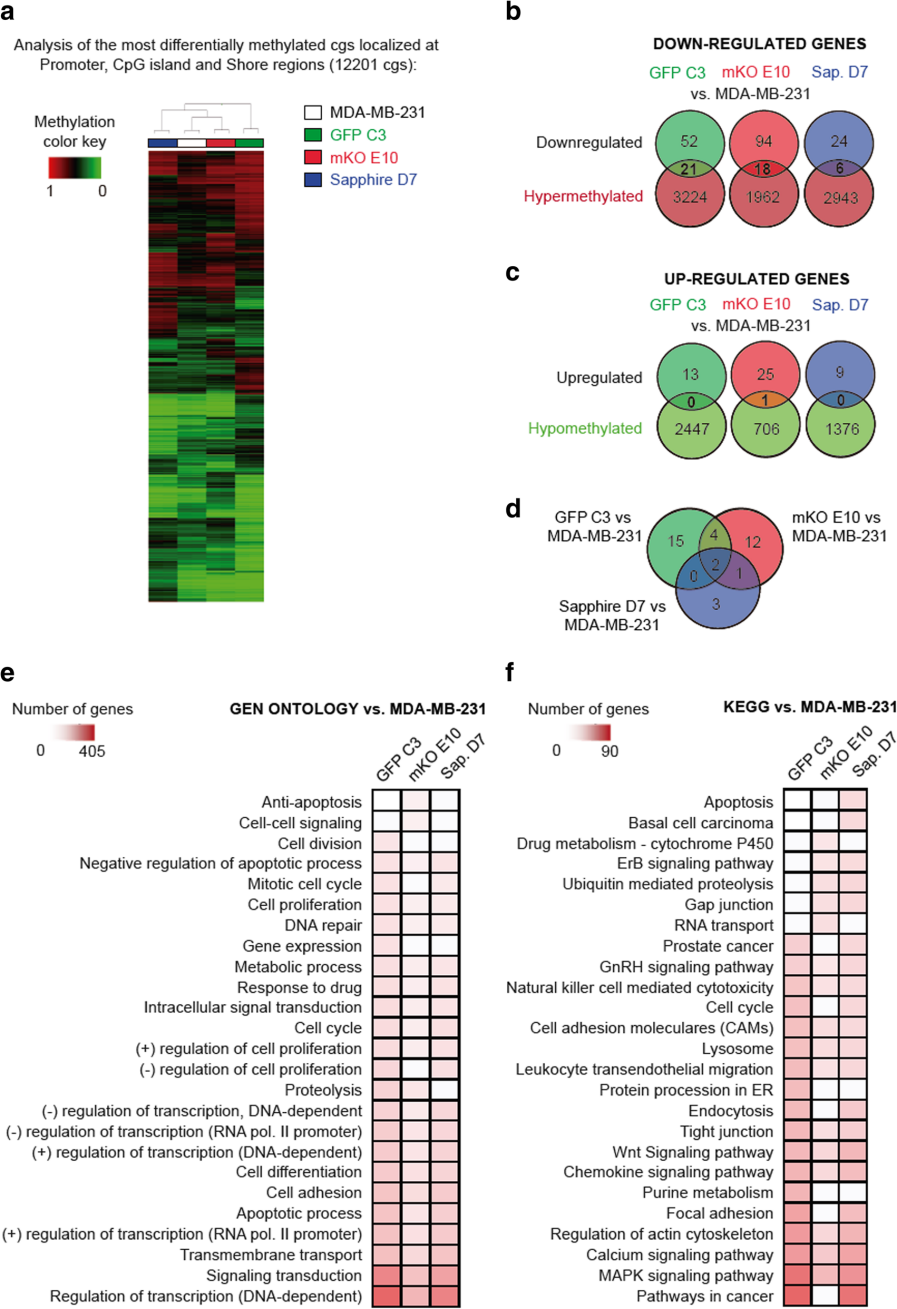
DNA methylation profile of MDA-MB-231 clonal cell lines. **a** Heatmap of the most differentially methylated CpGs (delta ≥0.20) localized at promoter, CpG island and shore regions (12,201 CpGs). **b-c** Identification of genes whose expression changes could be explained by methylation differences: (b) downregulated vs hypermethylated and (c) upregulated vs hypomethylated. **d** Venn diagrams of methylation gene changes of selected clonal cell lines vs. MDA-MB-231. **e-f** Gene Ontology (e) and KEGG (f) with differentially methylated genes (delta ≥0.20) at promoter and CpG island and shore

Epigenetic mechanisms, such as DNA methylation, are important regulators of gene expression. To establish if the observed changes in gene expression were due to DNA methylation modifications, we determined the genomic methylation pattern of the individual clones and the parental cell line (Fig. 5). To extrapolate data from these measurements that are probably responsible for the observed changes in gene expression, we focused our analysis on CpG islands and shore regions of gene promoters. The most differentially methylated CpGs in these regions are shown in the heatmap in Fig. 5a. The comparative analysis of the methylation signature revealed specific, characteristic methylation patterns for the individual clones and for the parental cell line MDA-MB-231. To determine if these changes caused the observed alterations in gene expression, we analyzed the association between gene expression levels and the methylation status of their promoter regions. As represented in Fig. 5b-c, changes in the methylation patterns were linked to discrete changes in the expression of the respective genes. Downregulated gene expression correlated to hypermethylation of the promoter in 40% (GFP), 19% (mKO) and 25% (Sapphire) of cases (Fig. 5b). We also observed changes in gene expression that were common to the different clonal cell lines. Four altered methylation patterns could be linked to similar changes in gene expression in two of the clonal cell lines, and two altered methylation patterns were linked to gene expression changes in all three clones (Fig. 5d).

In summary, the observed alterations in gene expression patterns could indeed be partly linked to different epigenetic states of the cell. Notably, changes in the methylation patterns often affect the expression of genes that act upstream of signaling cascades. It is therefore plausible to assume that changes in the methylation pattern of core regulatory genes may play a crucial role in the observed alterations in gene expression (Fig. 5e-f).

#### Cytokine arrays

**Fig. 6:**
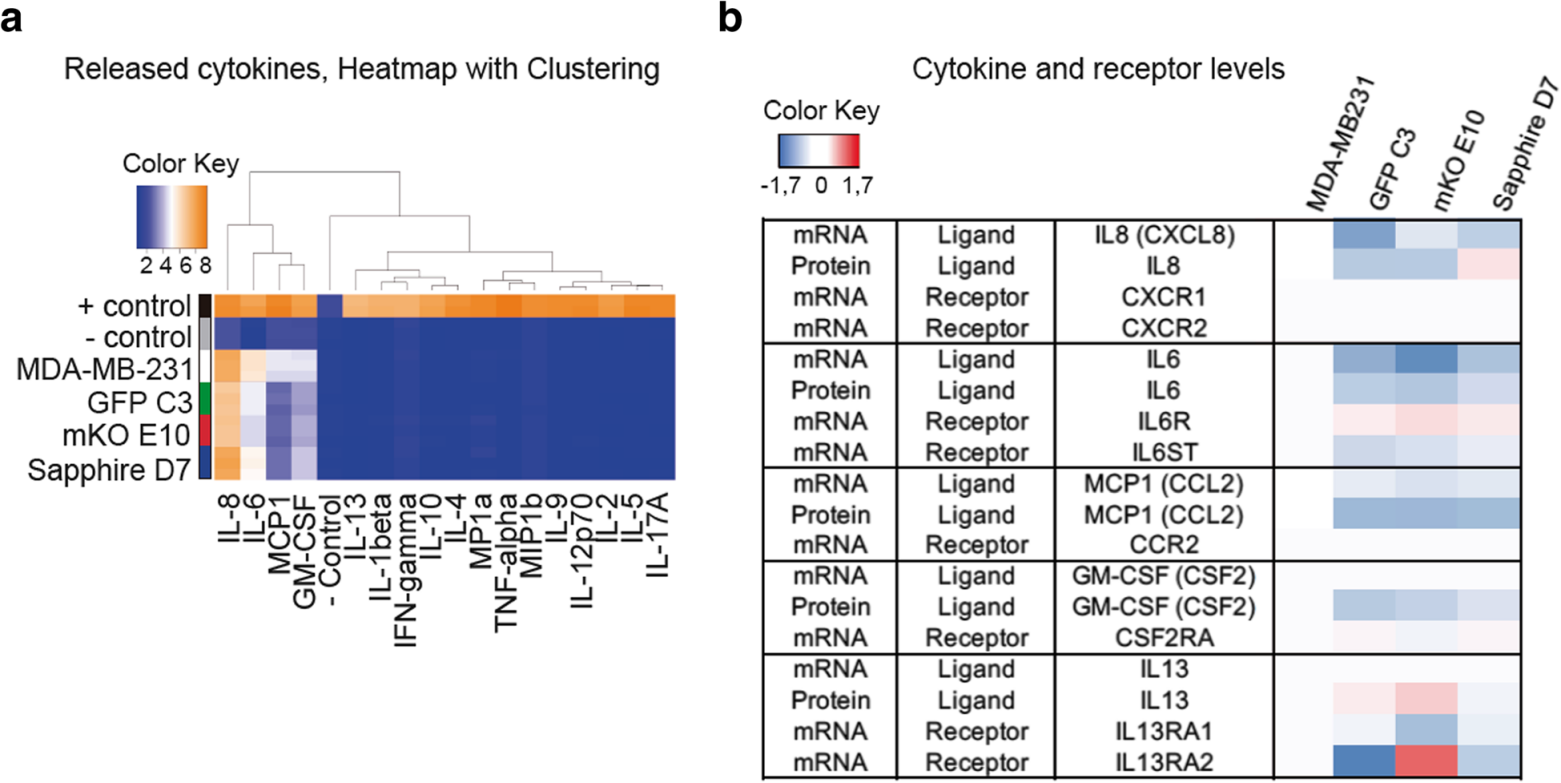
Cytokine expression. **a** Heatmap with clustering of released cytokines from the previous clonal cell lines. **b** Released cytokine mRNA and protein levels vs receptor levels

To confirm these alterations of cytokine-mediated signaling pathways at the protein level, we used cytokine arrays to analyze the composition of secreted factors in the individual clones (Fig. 6a). Among the 17 key cytokines tested, quantifiable amounts were detected for IL8, IL6, MCP1(CCL2), GM-CSF and IL13. Among those, MCP1 was reduced more than 10-fold in all clonal cell lines compared to the parental cell line. Weaker but still significant alterations were detected for GM-CSF and IL6. Clone-specific differences were detected for IL8, which was strongly reduced in the clones mKO E10 and GFP but unaltered in the clone Sapphire, and higher levels of IL13 were detected in the GFP C3 and mKO E10 clonal cell lines. To complete this analysis, we merged two datasets (cytokine expression and mRNA receptor expression) to elucidate which cytokine-cytokine receptor interactions had observable changes between the parental cell line and the different clonal cell lines (Fig. 6b). This comparison revealed that cytokine-mediated signaling pathways might be affected by altered expression of cytokine receptors, amount of cytokines released, or by simultaneous changes in cytokines and cytokine receptors.

### Interplay between the different clones

#### Co-culture experiments

**Fig. 7:**
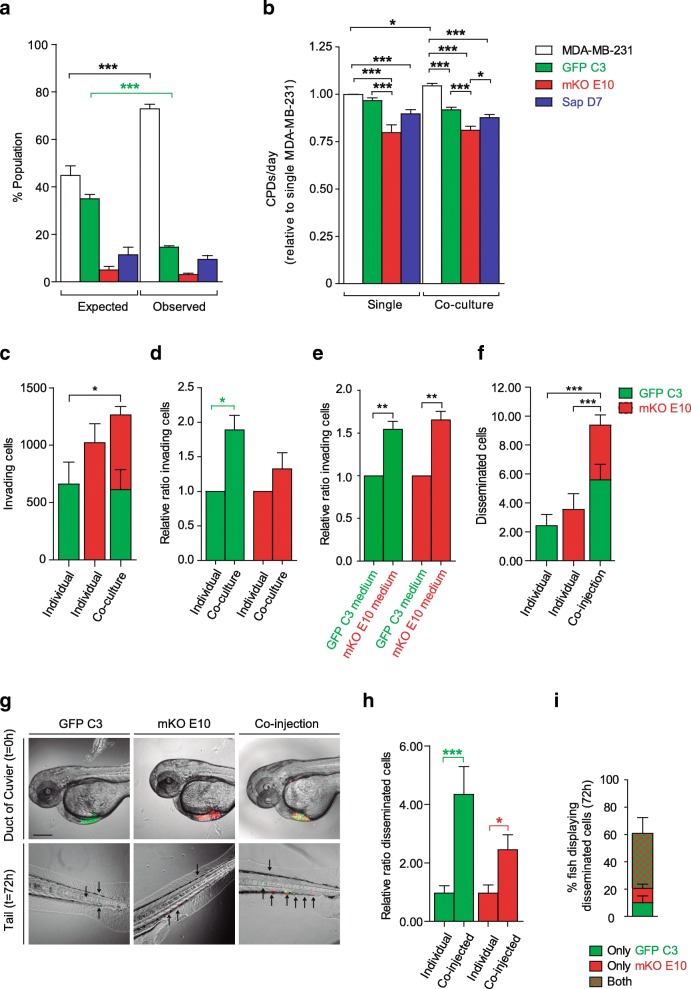
Clone interactions among parental and clonal cell lines in co-culture for 28 days **(a-b). a** Expected population percentage of total population calculated using cumulative population doublings (CPDs) per day vs. observed population percentage. **b** CPDs/day of single culture vs. CPDs/day of the same cell line in co-culture. **c-e** Cooperation of GFP C3 and mKO E10 in invasion assays: (c-d) Evaluation of cooperation on the invading capacity of single clones vs co-cultured clones**:** (c) Total number of invading cells and (d) Relative ratio of invading cells based on the cell number per clone cultured individually. (e) Invading capacity of cells conditioned with their own medium or medium from another clonal cell line. Relative ratio of invading cells based on the cell number per clone cultured with their own conditioned medium**.** (**f-i**) In vivo clonal cooperation. Cooperation of GFP C3 and mKO E10 in in vivo migration assays (Zebra fish model). Disseminated cells from the duct of Cuvier to the tail 72 h after injection: individual injection of GFP C3 and mKO E10 and co-injection of both clonal cell lines: (f) Total number of disseminated cells, (g) representative graph (scale bar = 70 μm), (h) relative ratio of invading cells based on the initial cell number per clone, (i) analysis of the coexistence of clones in the co-injected fish: percentage of co-injected fish with GFP C3 cells only, mKO E10 cells only, or both. Significant differences were determined using unpaired t-test with Welch’s correction. Asterisks indicate significant differences when P-values are < 0.05 (*), < 0.01 (**), and < 0.001 (***)

Co-culture experiments were performed to test if the cooperation between different clones might affect the growth rate of the individual clones. MDA-MB-231, GFP C3, mKO E10 and Sapphire D7 cell lines were cultured individually and in different combinations for 28 days (Fig. 7, Additional file 5: Figure S4a-c). In line with the results obtained in the in vivo mouse model (Fig. 2e), the mKO clonal cell line was progressively reduced and after 28 days of co-culture was severely depleted, representing just 3% of the total population (Fig. 7a, Additional file 5: Figure S4a). The decrease of mKO cells when mixed with the parental cells may indicate that its growth is overtaken by that of other clones. This result, and the result for Sapphire D7, matched the expected values determined by the calculated cumulative population doublings (CPDs) from culture of the individual cell lines (Fig. 7b). In contrast, the percentage of cells from the clone GFP C3 was significantly lower than the expected values (Fig. 7a). The proliferation rate of MDA-MB-231 and GFP C3 was not significantly different in single culture (Fig. 1b); however, the increase of CPDs for MDA-MB-231 in co-culture (Fig. 7a) induced a significant decrease of the clone GFP C3 in the total population (Fig. 7b). After 28 days in culture, the percentage of MDA-MB-231 in the population was 28% higher than expected, while the observed percentage of GFP C3 (14.72%) was lower than expected (39.94%) (Fig. 7a). These results were confirmed in experiments in which only the parental cell line and the clone GFP-C3 were combined (Additional file 5: Figure S4b-c). In all combinations involving GFP C3, the observed percentages of MDA-MB-231 increased and GFP C3 decreased.

#### Resistance to stress in co-culture experiments

We show in Additional file 5: Figure S4b how the presence of clonal cell lines with slower proliferation in the total population tends to decrease as time in co-culture increases. In the absence of stressors, such cells would be outcompeted after several weeks in co-culture. We aimed to test why these slower-proliferating cells were still present when we sorted the parental cell line. We hypothesized that cells could persist in the population was subject to a stressor that provided any benefit to the slower cell line or any disadvantage to the faster-proliferating cell lines. In Additional file 5: Figure S4d we show that mKO E10 was the clonal cell line most resistant to arsenite treatment, followed by GFP C3 and Sapphire, MDA-MB-231 being the least resistant. We wondered how the co-cultured population would evolve in the presence of this stressor. Additional file 5: Figure S4e-f shows how the percentage of clones (GFP C3 or mKO E10) in co-culture was significantly higher (and consequently, the percentage of MDA-MB-231 cells was lower) in the presence of arsenite than in the absence of arsenite. The clone population increases because they are more resistant than MDA-MB-231. We have included this as an example of a stressor, but other stressors could also affect the resistance of the clones, for example, resistance to growth from a single cell (subcloning). This allows us to explain why slower-proliferating clones were present in the MDA-MB231 cell line when we selected them, as they could be more resistant to a particular stressor than the whole population, and thus persist. Therefore, our results show that maintenance in the total population it is not only dependent on the proliferation rate; environmental conditions can affect the presence of the clones.

#### Clonal cooperation on migration and invasion assays

In addition to clonal cooperation by secreted factors, cells of a heterogenous tumor might use feature complementation to achieve complex cellular processes like invasion into the cellular matrix – for which not only cell migration, but also digestion of the extracellular matrix and chemotactic abilities are required.

To test if our cell line model could reproduce such a complementary behavior between different clones, we tested the invasive capacity of the phenotypically more distinct clones, mKO E10 and GFP C3, alone and in combination in the Matrigel invasion assay (Fig. 7c-d). Comparison of the total number of invading cells revealed that cells in the clone mixture were significantly more effective at invading than the cells from the clone GFP C3 alone (Fig. 7c). To estimate the contribution of each clone to this increased invasive capacity, we must take into consideration that although the total number of seeded cells was the same as when we seeded a single clone (30,000 cells), the number of cells from each clone in the mix represents only 50% of the cells when seeded individually (15,000 GFP C3 cells + 15,000 mKO E10 cells). Normalization of the data revealed that the invasive capacity of the clone GFP C3 increased two-fold when seeded together with the clone mKO E10 (Fig. 7d).

To determine if the increased invasion was the result of physical interactions among clonal cell lines or secreted stimulatory factors, we performed the invasion assay with GFP C3 and mKO E10 clonal cell lines seeded individually, adding complemented medium under the insert. We compared the invasion efficiency in the presence of their own complemented medium or medium complemented by the other clonal line, and found that invasion also increased in the presence of medium complemented by the other cell line (Fig. 7e).

To confirm these data in a more physiological context, we performed an assay to measure cell dissemination in the tail of zebrafish. Cells of the different clones were injected alone (300 cells) or in combination (150 GFP C3 cells + 150 mKO E10 cells) into the duct of Cuvier and the number of cells in the tail after 72 h was determined (Fig. 7f-i). We found that co-injection of both clonal cell lines resulted in significantly more cells in the tail of the fish than injection of individual clonal cell lines (Fig. 7f-g). In addition, a significant increase in the disseminating capacity of both clones was observed (four-fold GFP and two-fold mKO) when the number of injected cells was normalized for the respective conditions (Fig. 7h). The efficiency of the injection did not show significant differences between individual and co-injected cells (Additional file 5: Figure S4g), excluding injection efficiency as the cause of the differences in invasion. Determination of the number of fish that contained either both or only one of the clones after co-injection revealed that 40% of fish had a combination of GFP C3 and mKO E10, 9.95% had GFP C3 only and 10.29% had mKO E10 only (Fig. 7i), confirming that communication between the cells of the different clones and physical interaction account for the positive effect in dissemination.

#### Effect of secreted factors on cell growth

We aimed to determine if cell growth could be modified by secreted factors.

##### Cell growth in the presence of conditioned medium

**Fig. 8:**
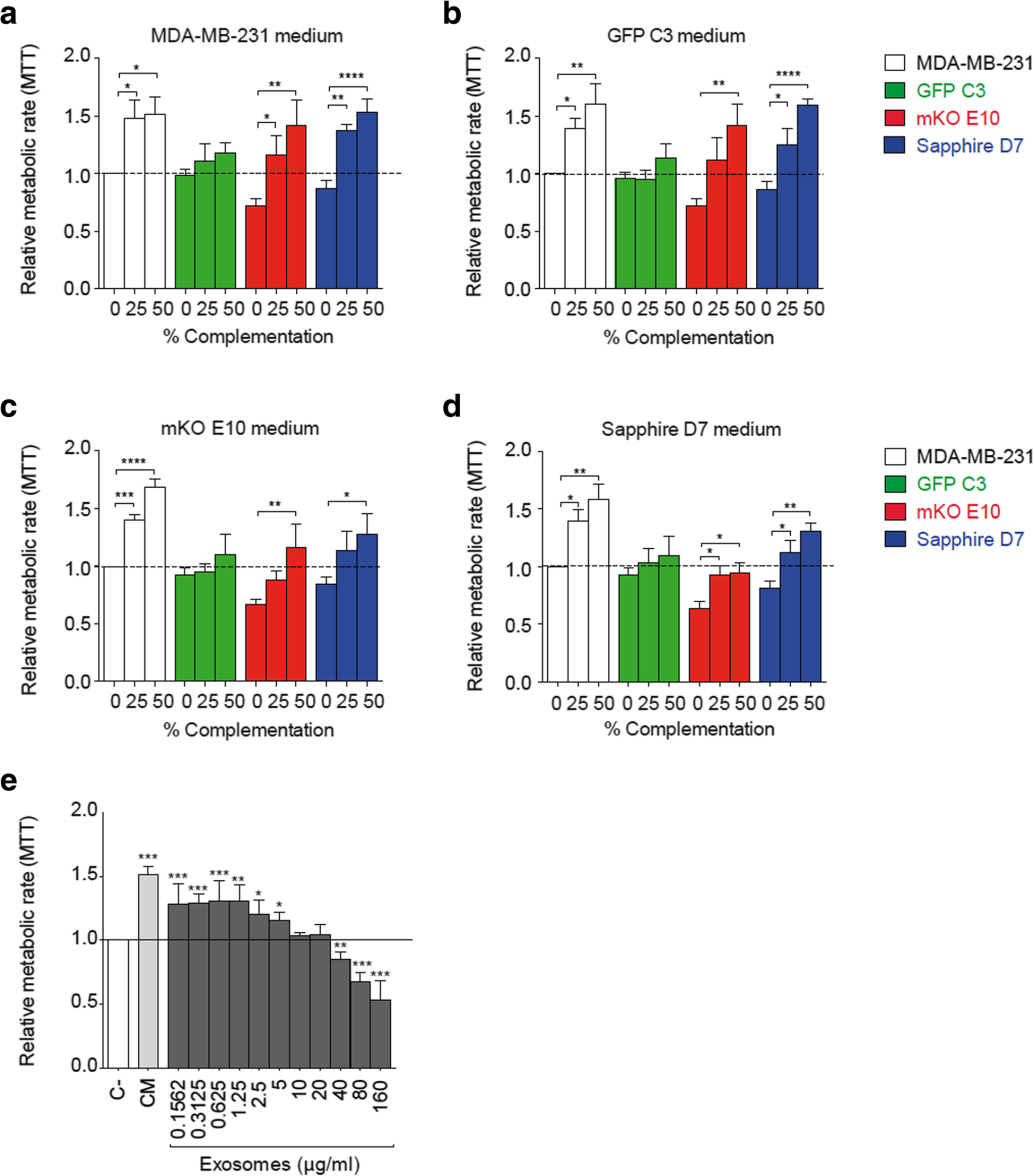
Clone communication by released soluble factors. **a-d** Metabolic activity by MTT assay of clonal cell lines, 72 h after medium complementation with supernatant extracted from MDA-MB-231, GFP C3, mKO E10 and Sapphire D7 cell line. Each graph corresponds to a cell line donor medium, (a) MDA-MB-231, (b) GFP C3, (c) mKO E10 and (d) Sapphire D7. Color bars indicate the receiving cell line. **e** Metabolic activity measurement of MDA-MB-231 by MTT assay after complementation with non-complemented medium (C-), 50% complemented medium (CM) and exosomes. Significant differences were determined using unpaired t-test with Welch’s correction. Asterisks indicate significant differences when P-values are < 0.05 (*), < 0.01 (**), and < 0.001 (***)

To study the effect of secreted factors (including cytokines and vesicles), conditioned medium (cell culture supernatant obtained after 96 h of culture) was applied in different concentrations to the individual cell lines and the growth rates were determined by MTT. The growth of the clonal cell lines mKO E10 and Sapphire D7 as well as the parental cell line increased with the conditioned medium from all other cell lines in a dose-dependent manner (Fig. 8a-d). These results therefore suggest that paracrine signaling promotes cell growth (Fig. 8a-d). While this growth-supporting effect of conditioned medium has long since been known, it was interesting that the clonal cell line GFP C3 did not significantly respond to any of the applied media. We could suppose that GFP C3, as the faster clonal cell line, does not require additional supplements from other cell lines. The consequence of this lack of response is shown in co-culture with cells with a similar growth rate. In Fig. 7a and Additional file 4: Figure S4b-c we see how the GFP C3 population decreases faster in the presence of MDA-MB231, comparing the percentage of observed populations (experimentally) and expected population (calculated from proliferation rate when the clonal cell line is cultivated individually). We observed this phenomenon when MDA-MB231 and GFP C3 were cultured together and in the presence of a third and even a forth clonal cell line. As we have shown, GFP C3 proliferation does not increase with medium complementation from other cell lines, however all the other cell lines do (Fig. 8a-d). The lack of response of GFP C3 to medium complementation can modify the expected evolution of the clonal composition: instead of being maintained at the same percentage as MDA-MB-231 (expected), GFP C3 is progressively reduced.

##### Extracellular vesicle induced cell growth

To identify factors responsible for the increased metabolic rates upon treatment with conditioned medium, we isolated extracellular vesicles from the conditioned medium by ultracentrifugation. We confirmed that all the clonal cell lines were able to release factors that increase metabolic rate (Fig. 7a-d) and that selected clonal cell lines were also able to release exosomes (Additional file 6 Figure S5). We selected the parental cell line as the exosome donor based on our previous results: (1) analysis of vesicle size and protein composition did not reveal major differences in the vesicles isolated from the parental cell line and the individual clones (Additional file 6: Figure S5a-e) and (2) cell lines responded positively to the addition of the factors, independently of the donor (Fig. 8a-d). Isolated extracellular vesicles from the parental cell line were then added to the parental cell line and the effect on cell growth was determined. Addition of increasing amounts of vesicles to the cells modified the metabolic rate of the receiving cells (Fig. 8e). The maximal increase in metabolic activity was observed with concentrations up to 1.25 μg exosome protein/mL. Higher concentrations of vesicles decreased the effect on metabolic activity. Thus, extracellular vesicle fractions contain factors that can modify the metabolic activity of the receiving cells in a dose-dependent manner.

## Discussion

Breast cancer is the most frequently diagnosed neoplasm in women and a leading cause of cancer death [1, 2]. Intertumor heterogeneity is one of the greatest issues in oncology, but it is not the only factor that makes patient treatment difficult: variation within a single tumor – intratumor heterogeneity – is also a key factor in determining the best treatment [51].

Cellular heterogeneity in established breast cell lines, including the MDA-MB-231 cell line, has been described previously [45, 52]. In this study we corroborate clonal heterogeneity in the MDA-MB-231 cell line of breast carcinoma, after the selection of clones marked with fluorochromes, using the StarTrack system (Additional file 1: Figure S1c). Clonal cell lines were selected to study the relevance of clonal communication in tumoral heterogeneity. The results from the experiments in vivo and in vitro demonstrated that despite all the observed phenotypic differences, the parental cell line, composed of a mix of an undetermined high number of clones, was equal or superior in terms of malignancy (tumor composition and metastasis) to the individual clonal cell lines in all tested conditions. Because the parental cell line is a mix of different clones, these results led us to the hypothesis that the interplay between different clonal populations within a heterogeneous population, by intercellular communication and/or feature complementation, provides advantages over isolated clonal cell lines.

Our results show that the mix of three clonal cell lines did not recover the features of a multiclonal population. For the study of tumor heterogeneity, the subcloning method limits the number of clones and makes reconstitution of a highly multiclonal population difficult. Therefore, the mix of several clones cannot be equated to a multiclonal population; rather, a parental cell line must be included to provide a true multiclonal population. However, subcloning may be a suitable method for the independent study of the single clones that constitute a cancer cell line or tumor.

Selected clonal cell lines showed variations in cell morphology (Fig. 1a, Additional file 2: Figure S2a), just as pathologists observe variation between different regions of the same tumor [53, 54]. The clonal cell lines also showed differences at the biological (Fig. 1), tumorigenic (Figs. 2, 3, Additional file 3: Figure S3) and molecular levels, and in gene expression (Fig. 4), DNA methylation pattern (Fig. 5) and cytokine expression (Fig. 6), with clear functional heterogeneity. Parental cells showed a faster growth rate (Fig. 1b-e) and biological aggressiveness – a greater presence in metastases – than the clones we studied (Fig. 3, Additional file 3: Figure S3). Our results show that the phenotypes of the single derived clones were heritable in the conditions used in this study; however, culturing subclones for a longer time could induce phenotypic changes if molecular or epigenetic alterations accumulated. In such a situation, heritability could be tested by studying the phenotype of sub-clones derived from the original clones, having cultured the original clones for several months.

Moreover, in co-culture (Fig. 7, Additional file 5: Figure S4a-c) the parental cells grew faster than the single clones, indicating that the parental cell line harbors a sum of clones that may outperform single clones through local or secreted factors, as yet not identified. These findings support the concept of *the whole is greater than the sum of its parts* and describe a model where the interplay of clones confers aggressiveness, and which may allow the identification of factors involved in cellular communication and metastasis. Thus, clonal heterogeneity allows the malignant cell line to acquire the greatest malignant potential.

Conventional models propose that each metastasis originates from a single tumor cell [55–57]. However, recent studies using mouse models of cancer have demonstrated that multiple subclones undergo polyclonal seeding and demonstrate interclonal cooperation between multiple subclones [7, 58]. Our results confirm (Fig. 3d) that metastasis could be formed either by a single (Fig. 3e) or several clonal cell lines (Fig. 3d). However, even in cases of metastasis formed by several clonal cell lines, each metastasis contained one predominant clone (Fig. 3d). Comparative studies indicate monoclonal patterns of seeding, suggesting that clones compete to metastasize. However, polyclonal seeding, in which multiple clones from the primary tumor seed the same metastasis, is also observed, indicating subclones might cooperate as well as compete to metastasize [7, 59]. In our model the cells were injected as a mix of single cells, therefore the metastasis formed by more than one cell line originated from several cells that reached the lung together, demonstrating that the cells physically interact to form the metastasis.

Several studies call into question the theory of clonal progression by the progressive accumulation of genetic alterations and selection of more aggressive clones, supporting instead the proposed theory of clonal cooperation between tumor clones [20–23, 25–27]. Tumor multiclonality is also supported by the field cancerization theory [60, 61], which states that there are many genetic alterations in the normal tissue surrounding tumors that can give rise to independent clones. Similarly, supporting interpretations can be drawn from the stem cell hypothesis, as diverse clones can derive from more than one pluripotent stem cell [62, 63], and the Big Bang model of colorectal tumor growth where the tumor grows predominantly as a single expansion populated by numerous intermixed subclones [64]. Clonal cooperation has recently been suggested in studies of single cell sequencing [62, 65, 66]. The present study further supports the idea that there are several clones that together confer the properties of malignancy, thus strengthening the concept of clonal cooperation, whereby clones synergistically provide certain selective advantages for proliferation, resistance to apoptosis, induction of angiogenesis, and interaction with environmental factors and inflammatory cells [20, 21, 30, 63].

Our results show that cells are able to interact and the coexistence of clonal cell lines resulted in a positive effect (Fig. 7c-h). We could conclude that physical interaction between clones (Fig. 7c-h) and secreted factors (Fig. 7e, Fig. 8a-e) favored the tumorigenic capacities of the cells. Cells are able to secrete factors (Fig. 6a) and send messages to surrounding and distant cells (paracrine signaling). Studies of cellular communication in breast cancer have demonstrated tumor cells’ abilities to secrete factors increasing breast cancer cell proliferation and metastasis [67, 68].

Recently, interesting discoveries have been made in the field of paracrine signaling. Extracellular vesicles (EVs) have been postulated as a highly efficient method to transform cells. Several groups have demonstrated that EVs upregulate prometastatic and tumor angiogenesis pathways [69], even transforming normal cells into cancer cells [70, 71]. Our complementation assay confirmed intercellular communication via factors released from the cells, but we could not distinguish between soluble factors (such as cytokines) or released EVs. Our results showed that exosomes were able to modify growth rate (Fig. 8e). However, the soluble factors seemed to be more efficient than EVs, as the effect of exosomes was dose-dependent.

Our results demonstrate how some clones, via soluble factors, EVs and physical interactions, are able to induce increased aggressiveness in other clones. Therefore, we can conclude that the Darwinian clonal progression theory should be complemented with a “cooperative model”, where clones are able to interact and transfer “properties” among one another.

In our study, the biological differences observed between the different clones have been corroborated by clear differences in the RNA expression arrays (Fig. 4), which were complemented with methylomic studies (Fig. 5). We have also demonstrated clonal communication (Figs. 7, 8), and the challenge now is to identify the factors released by the parental cells that enhance survival and metastasis. The identification of factors and cytokines that modulate cellular communication and enhance malignant properties will be essential to understand the formation of clonal clusters and prevent metastasis. We propose the term *functional heterogeneity* to highlight the different expression between clones due to extracellular factors and epigenetic changes. This approach opens the way to new paradigms in the development of metastases, including central factors at an intracellular level, and factors involved in the communication between tumor cells and microenvironmental or inflammatory cells.

In summary, these data support that functional clonal heterogeneity does not always reflect genetic heterogeneity. Inhibition of clonal cooperation, by blocking cytokines or other factors involved in the formation of clusters and the interplay with environmental cells may represent a change in the therapeutic paradigm to prevent the development of metastasis [21].

## Conclusions

Interplay between clones confers malignancy, supporting the concept of clonal cooperation, whereby clones synergistically provide certain selective advantages by physical interaction, soluble factors and extracellular vesicles.

## Additional files

Additional file 1: **Figure S1.** Generation of fluorescent clonal cell lines. **a.** Mechanism to induce the expression of fluorescent proteins by transposon integration. **b.** Fluorescent proteins codified by transposons. **c.** Representative images of the clonal cell lines (scale bar = 50 μm) obtained by transposon integration and subcloning: phase contrast and specific fluorescence for every clonal cell line. (PDF 994 kb)

Additional file 2: **Figure S2.** Phenotypic characterization of MDA-MB-231 GFP C3, mKO E10, and Sapphire D7 cell lines. a. Morphological evaluation by phase contrast images (scale bar = 200 μm). b. Cumulative population doublings per day (CPDs) (relative to MDA-MB-231 CPDs at 7 days in culture) at 7, 14, 21 and 28 days in culture. c-d. Migration capability by wound healing assay: measurements relative to MDA-MB-231 (c) and representative images (scale bar = 200 μm) (d). Significant differences were determined using ANOVA (Tukey’s multiple comparisons test) (b) and Tukey’s unpaired t-test with Welch’s correction (c). Asterisks indicate significant differences when *P*-values are < 0.05 (*), < 0.01 (**), and < 0.001 (***). (PDF 1639 kb)

Additional file 3: **Figure S3.** Homing capacity of parental, clonal cell lines and an equal mix of all cell lines. Analysis of lung metastasis. Hematoxylin/eosin staining. Complete lung reconstruction (scale bar = 400 μm). (PDF 823 kb)

Additional file 4: **Table S1.** Analysis of mKO mRNA expression vs MDA-MB-231 with the Human Cancer Metastasis Database. (PDF 511 kb)

Additional file 5: **Figure S4.** Clone interactions among parental and clonal cell lines: Co-culture for 28 days. **a**. Percentage of total population represented by clonal cell lines and MDA-MB-231 at 0, 7, 14, 21 and 28 days in co-culture. **b-c**. Expected (E) percentage of total population calculated using CPDs per day vs. observed (O) population percentage: (b) Co-culture of two clonal cell lines and (c) co-culture of three cell lines. **d-f**. Effect of arsenite on co-culture: (d) Proliferation 72 h after arsenite treatment (250 μM-90 min). (e-f) Percentage of cell line populations after arsenite treatment: (e) MDA-MB-231 vs GFP C3, (f) MDA-MB-231 vs mKO E10. The percentage of each cell line in the total population was detected at seeding (day 0), 90 min (day 1) and 72 h (day 4) after treatment. **g. Co-injection in zebra fish model:** percentage of fish with cells in the tail 72 h after injection with individual clones or co-injected with the mix. Significant differences were determined using Tukey’s unpaired t-test with Welch’s correction (b, c, d, e, f). Asterisks indicate significant differences when P-values are < 0.05 (*), < 0.01 (**), and < 0.001 (***). (PDF 917 kb)

Additional file 6: **Figure S5.** Clone communication by exosomes. **a-c**. Characterization of exosomes: (**a**) area, (**b**) perimeter and (**c**) diameter. Significant differences were determined using unpaired t-test with Welch’s correction. Asterisks indicate significant differences when P-values are < 0.05 (*), < 0.01 (**), and < 0.001 (***). **d**. Representative transmission electron microscopy image of exosomes. **e**. Immunoblot of exosome markers (TSG101 and CD81) and housekeeping gene (β-actin). (PDF 868 kb)

## Data Availability

All microarray data in this publication have been deposited in the NCBI Gene Expression Omnibus and are accessible through the GEO Series accession number GSE122008 (https://www.ncbi.nlm.nih.gov/geo/query/acc.cgi?acc=GSE122008).

## Abbreviations

ANOVA: ANalysis Of VAriance
ATCC: American Type Culture Collection
BCA: BicinChoninic Acid
CD81: Cluster of Differentiation 81
CM: Complete Medium
CPDs: Cumulative Population Doublings
DMBA: 2,4-DiMethoxyBenzAldehyde
DMEM: Dulbecco’s Modified Eagle’s Medium
DNA: DeoxyriboNucleic Acid
DoC: Duct Of Cuvier
DPBS: Dulbecco’s Phosphate-Buffered Saline
dpf: Days Post-Fertilization
eGFP: Enhanced Green Fluorescent Protein
Em: Emission
EVs: Extracellular vesicles
Ex: Excitation
FACS: Fluorescence-Activated Cell Sorting
FBS: Fetal Bovine Serum
FC: Fold Change
FDR: False Discovery Rate
GEO: Gene Expression Omnibus
GFP: Green Fluorescent Protein
GM-CSF: Granulocyte Macrophage Colony-Stimulating Factor
GO: Gene Ontology
HBS: HEPES Buffered Saline
hpi: Hours Post Injection
HRP: HorseRadish Peroxidase
i.m.p.f: Inoculation at Mammary Fat Pad
IL: Interleukin
IR: Inverted Repeat
ITRs: Terminal Repeat Sequences
JAK-STAT: JAnus Kinase/Signal Transducers and Activators of Transcription
KEGG: Kyoto Encyclopedia of Genes and Genomes
MCP1: Monocyte Chemoattractant Protein 1
MIP1: Macrophage Inflammatory Protein
mKO: Monomeric Kusabira Orange
MTT: 3-[4,5-Dimethylthiazol-25-Yl]-2.5-Diphenyl Tetrazolium Bromide
NCBI: National Center for Biotechnology Information
NOD: Nucleotide-binding Oligomerization Domain
PBS: Phosphate-Buffered Saline
PFA: ParaFormAldehyde
PTU: N-Phenylthiourea
PVDF: PolyVinylidene DiFluoride
rcf: Relative Centrifugal Force
RNA: RiboNucleic Acid
RT-PCR: Real-Time Polymerase Chain Reaction
SDS: Sodium Dodecyl Sulfate
SEM: Standard Error of the Mean
SF: Soluble Factors
STR: Short Tandem Repeat
TEM: Transmission Electron Microscopy
TGFR-1: TGF-beta receptor type-1
TNBC: Triple Negative Breast Cancer
TPA: 12-O-TetradecanoylPhorbol-13-Acetate
TSG101: Tumor Susceptibility Gene 101
w/v: Weight/volume
WHO: World Health Organization
WNT: Wingless-related iNtegration Site
WT: Wild Type
