# Supplementary figures and images for: The role of clonal communication and heterogeneity in breast cancer
Source: BMC Cancer. 2019 Jul 5;19:666. doi: 10.1186/s12885-019-5883-y (PMC6612119; doi:10.1186/s12885-019-5883-y)

**a**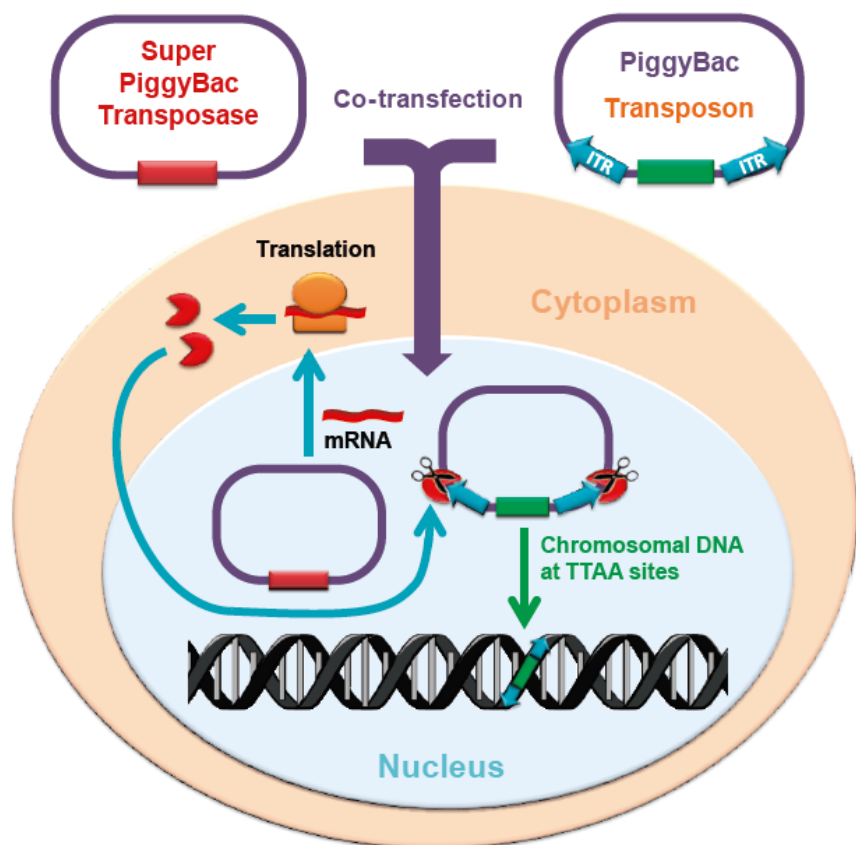**b**

**Fluorescent proteins  
codified by transposons**

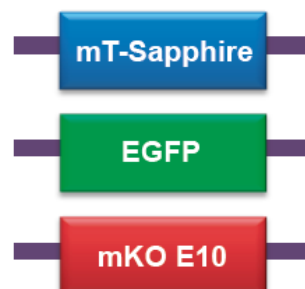**c**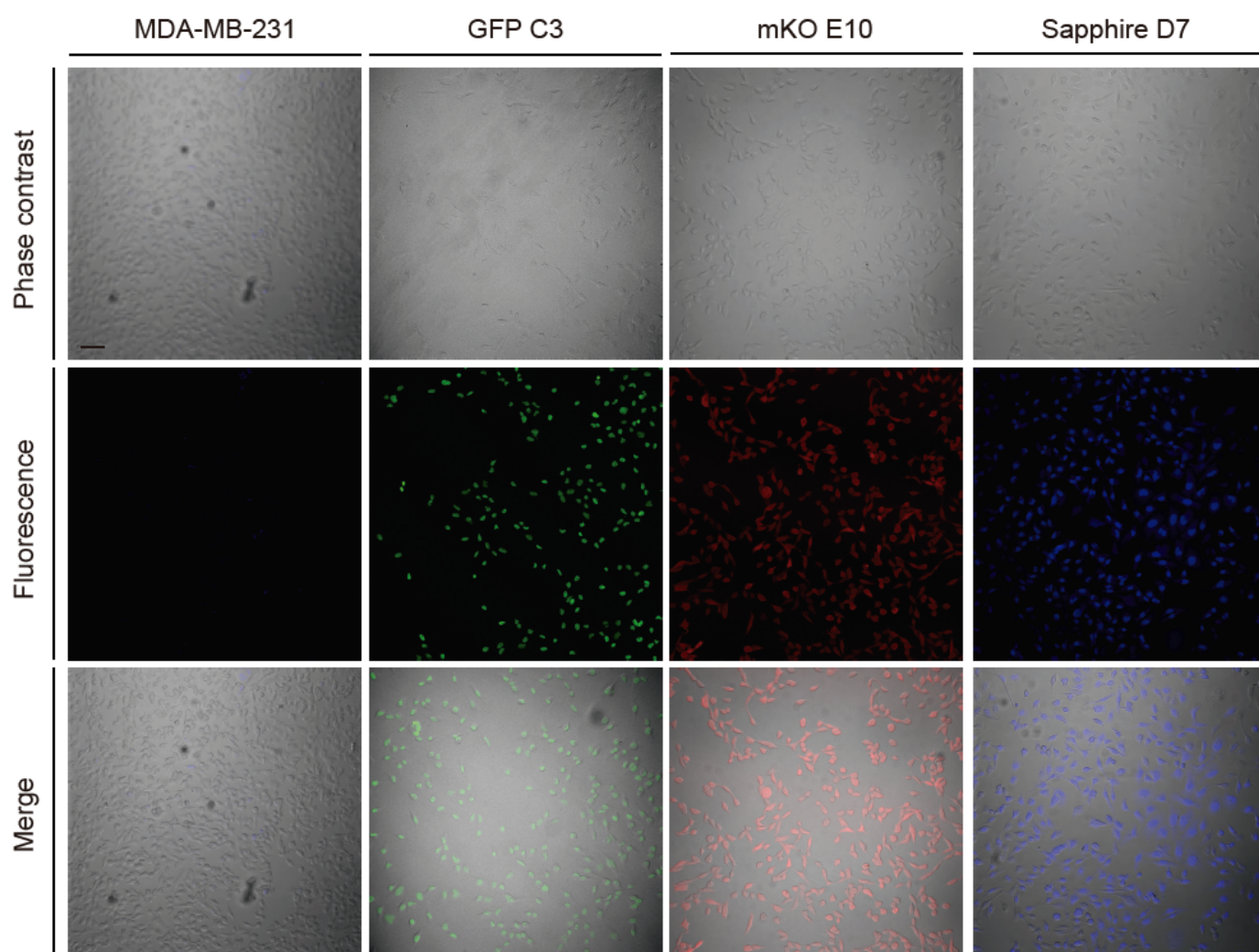

Supplement: Supplementary file 1 — Figure S1. Generation of fluorescent clonal cell lines. a. Mechanism to induce the expression of fluorescent proteins by transposon integration. b. Fluorescent proteins codified by transposons. c. Representative images of the clonal cell lines (scale bar = 50 μm) obtained by transposon integration and subcloning: phase contrast and specific fluorescence for every clonal cell line. (PDF 994 kb) [file 12885_2019_5883_MOESM1_ESM.pdf]

**a**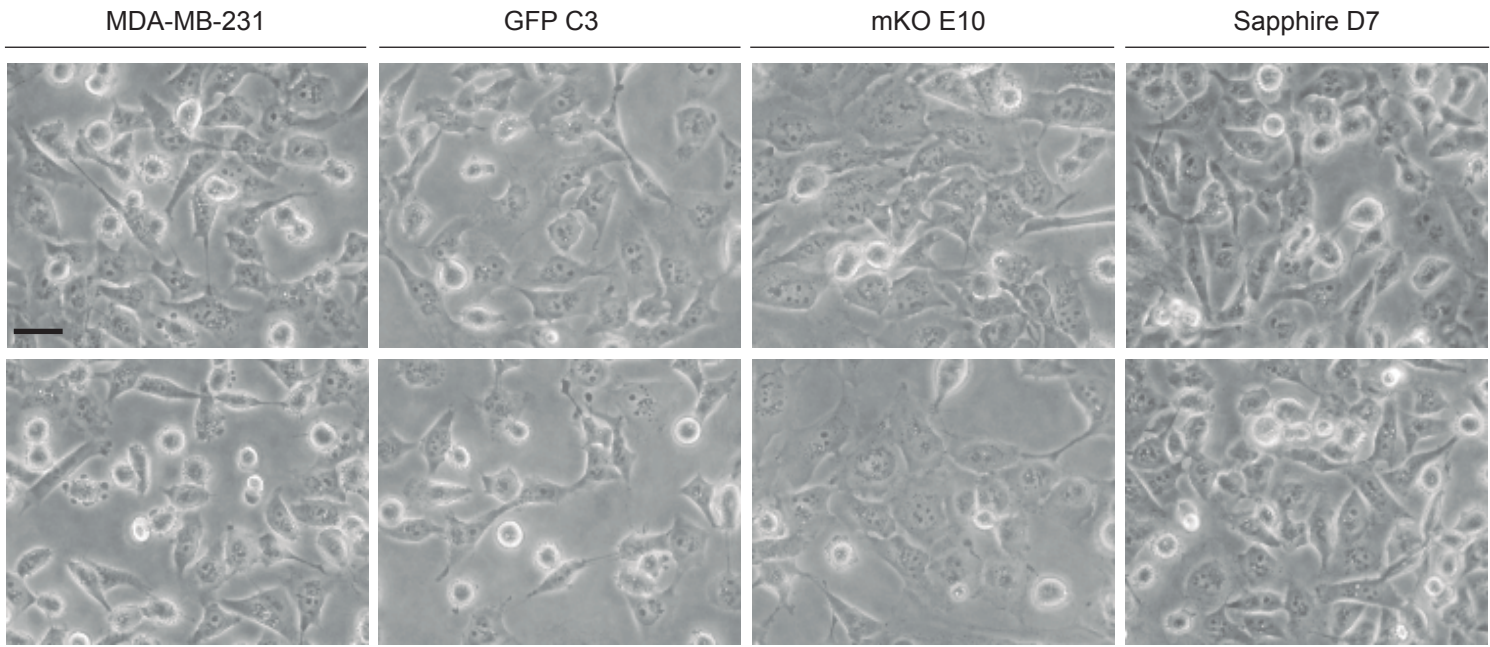**b**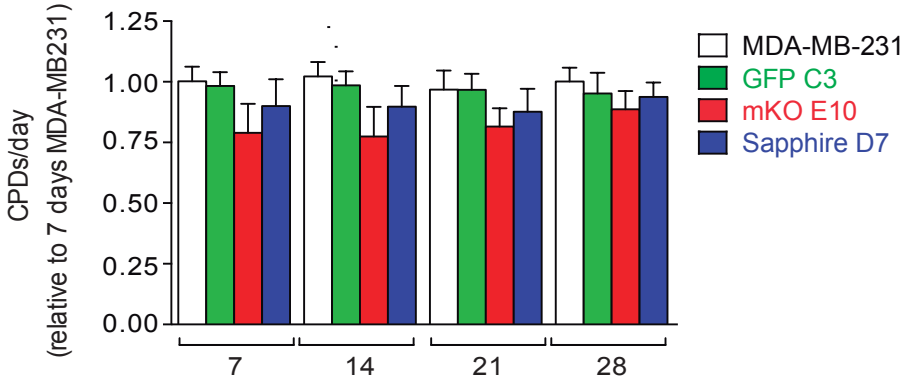**c**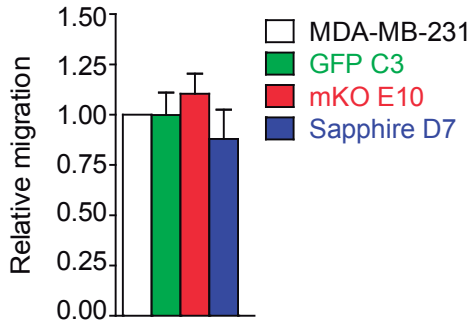**d**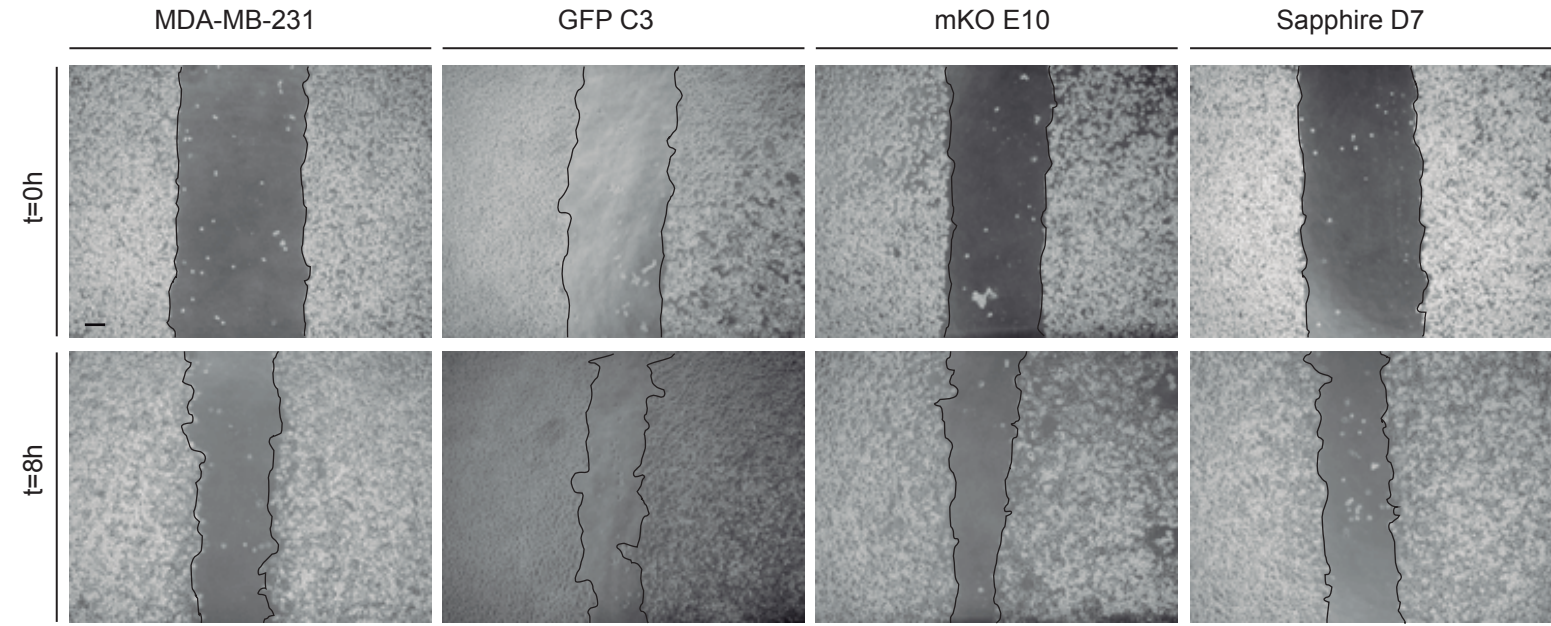

Supplement: Supplementary file 2 — Figure S2. Phenotypic characterization of MDA-MB-231 GFP C3, mKO E10, and Sapphire D7 cell lines. a. Morphological evaluation by phase contrast images (scale bar = 200 μm). b. Cumulative population doublings per day (CPDs) (relative to MDA-MB-231 CPDs at 7 days in culture) at 7, 14, 21 and 28 days in culture. c-d. Migration capability by wound healing assay: measurements relative to MDA-MB-231 (c) and representative images (scale bar = 200 μm) (d). Significant differences were determined using ANOVA (Tukey’s multiple comparisons test) (b) and Tukey’s unpaired t-test with Welch’s correction (c). Asterisks indicate significant differences when P-values are < 0.05 (*), < 0.01 (**), and < 0.001 (***). (PDF 1639 kb) [file 12885_2019_5883_MOESM2_ESM.pdf]

## Intravenous inoculation

| MDA-MB-231                                                                                      | GFP C3                                                                             | mKO E10                                                                             | Sapphire D7                                                                          | Mix                                                                                  |
|-------------------------------------------------------------------------------------------------|------------------------------------------------------------------------------------|-------------------------------------------------------------------------------------|--------------------------------------------------------------------------------------|--------------------------------------------------------------------------------------|
| <p>400 um</p> 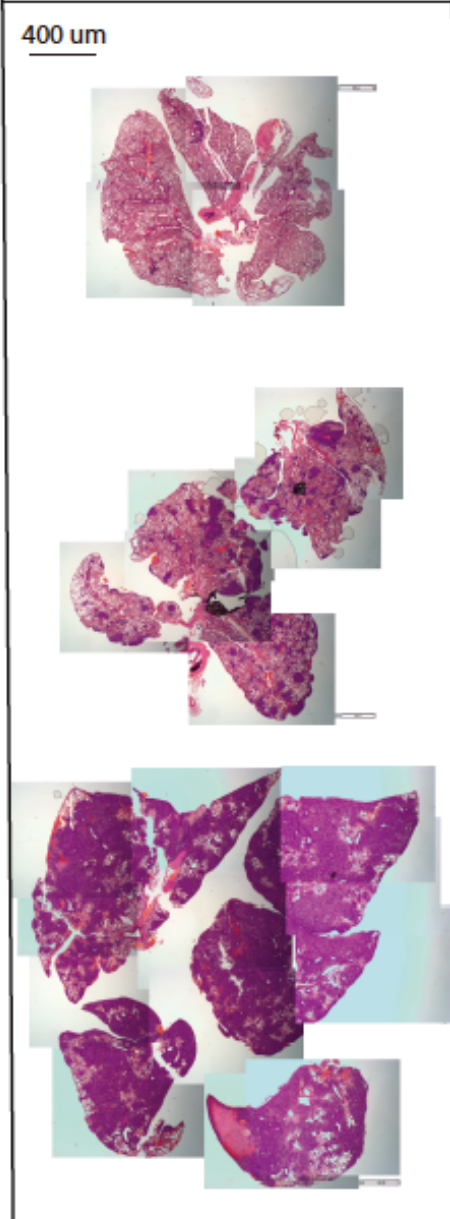 | 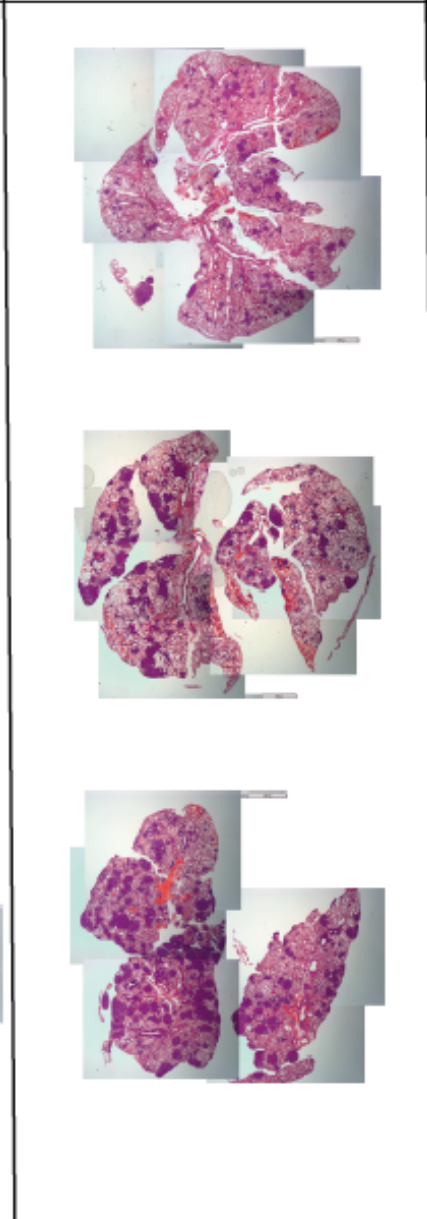 | 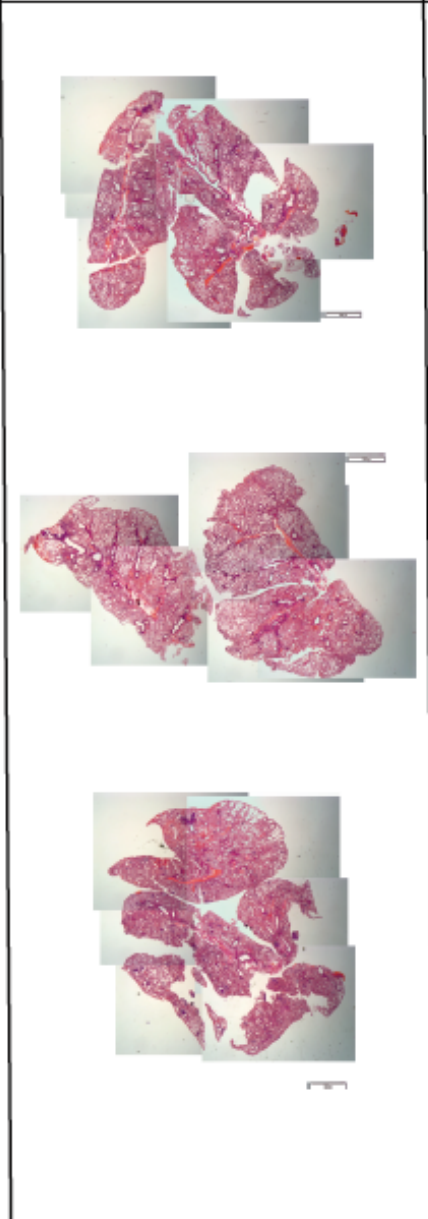 | 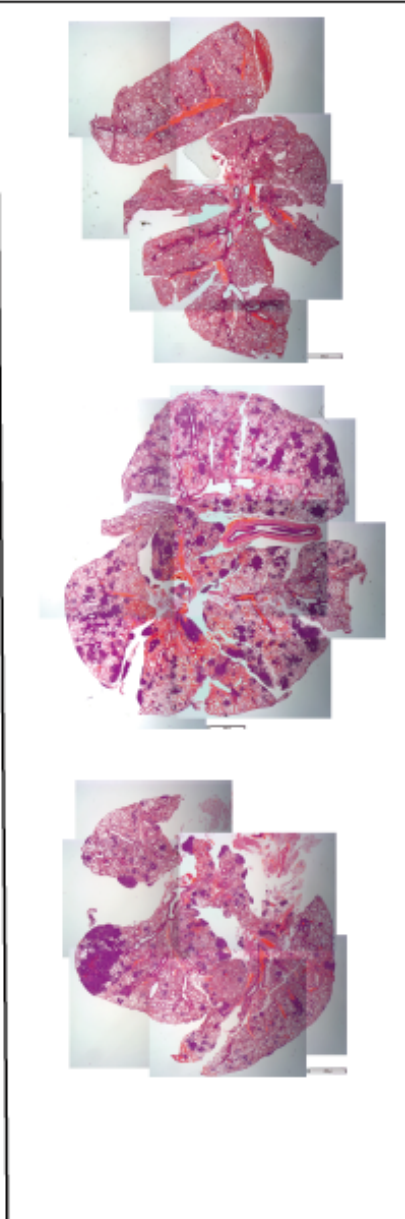 | 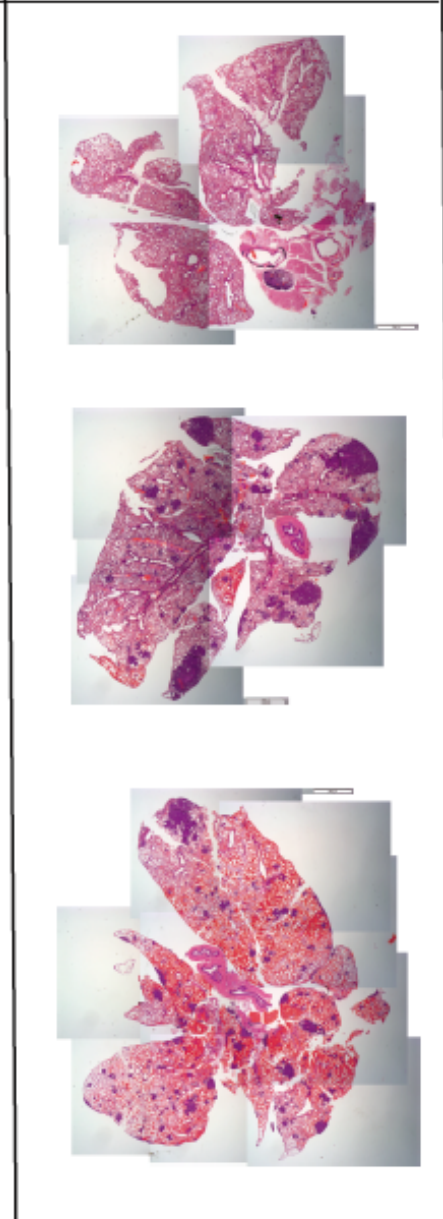 |

Supplement: Supplementary file 3 — Figure S3. Homing capacity of parental, clonal cell lines and an equal mix of all cell lines. Analysis of lung metastasis. Hematoxylin/eosin staining. Complete lung reconstruction (scale bar = 400 μm). (PDF 823 kb) [file 12885_2019_5883_MOESM3_ESM.pdf]

**a**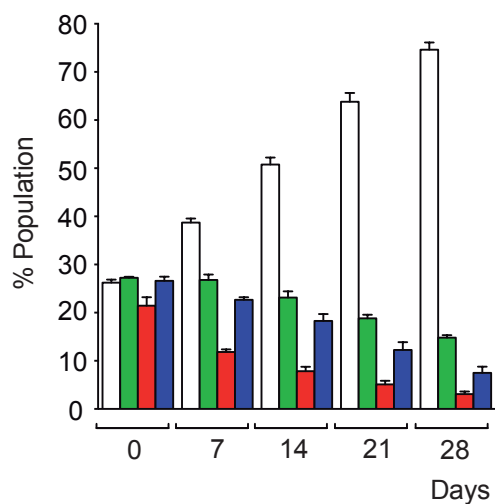**b**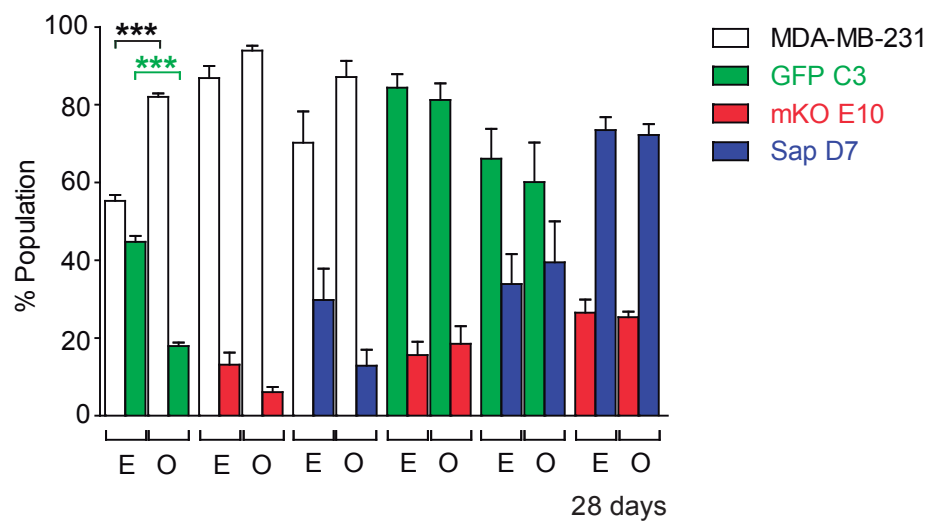**c**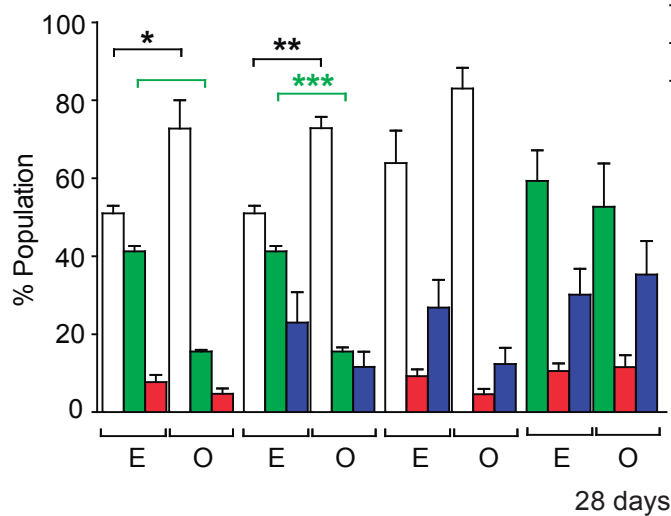**d**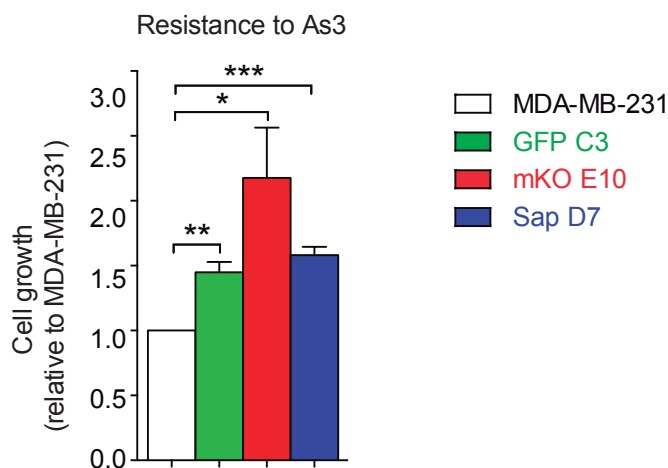**e**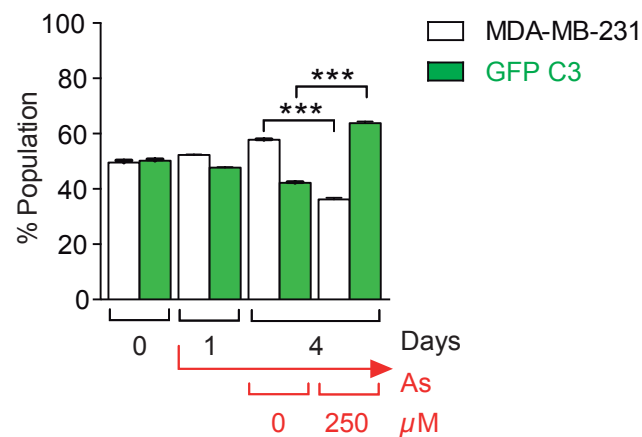**f**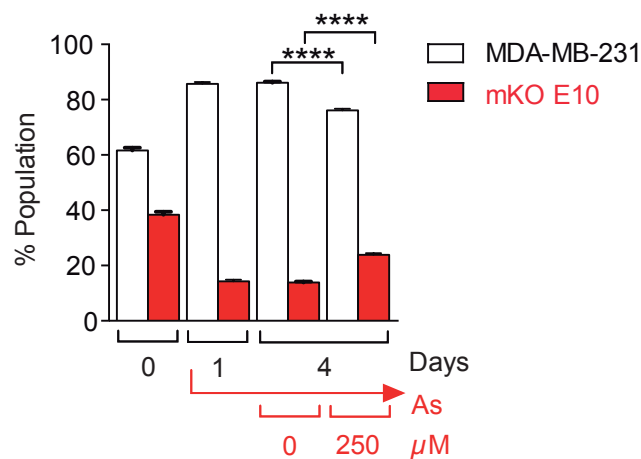**g**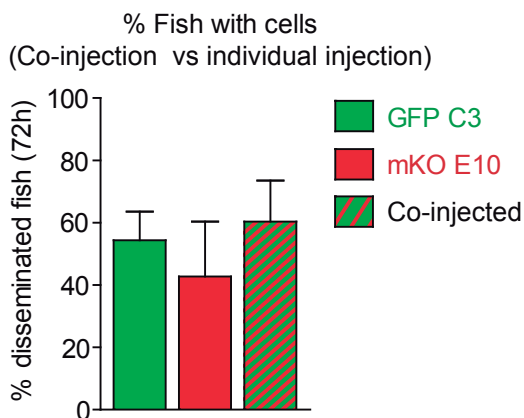

Supplement: Supplementary file 5 — Figure S4. Clone interactions among parental and clonal cell lines: Co-culture for 28 days. a. Percentage of total population represented by clonal cell lines and MDA-MB-231 at 0, 7, 14, 21 and 28 days in co-culture. b-c. Expected (E) percentage of total population calculated using CPDs per day vs. observed (O) population percentage: (b) Co-culture of two clonal cell lines and (c) co-culture of three cell lines. d-f. Effect of arsenite on co-culture: (d) Proliferation 72 h after arsenite treatment (250 μM-90 min). (e-f) Percentage of cell line populations after arsenite treatment: (e) MDA-MB-231 vs GFP C3, (f) MDA-MB-231 vs mKO E10. The percentage of each cell line in the total population was detected at seeding (day 0), 90 min (day 1) and 72 h (day 4) after treatment. g. Co-injection in zebra fish model: percentage of fish with cells in the tail 72 h after injection with individual clones or co-injected with the mix. Significant differences were determined using Tukey’s unpaired t-test with Welch’s correction (b, c, d, e, f). Asterisks indicate significant differences when P-values are < 0.05 (*), < 0.01 (**), and < 0.001 (***). (PDF 917 kb) [file 12885_2019_5883_MOESM5_ESM.pdf]

**a**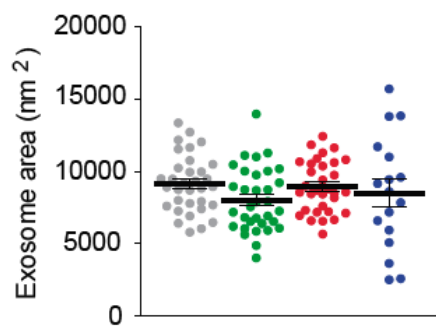**b**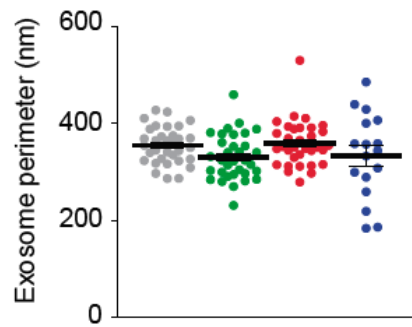**c**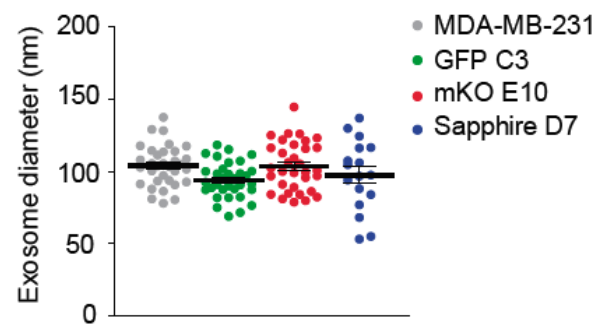**d**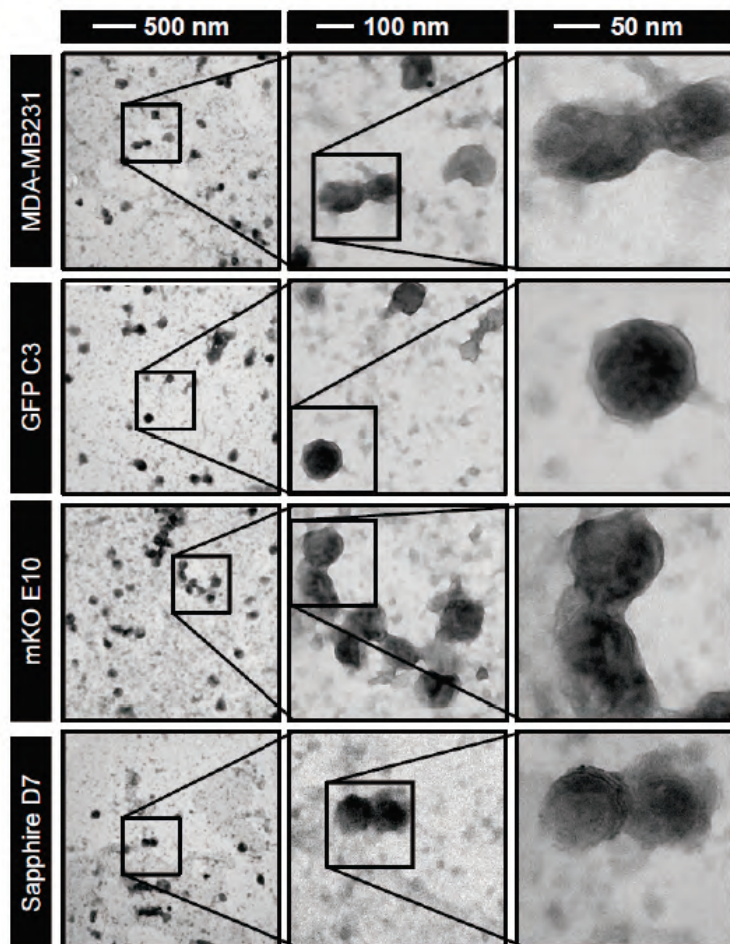**e**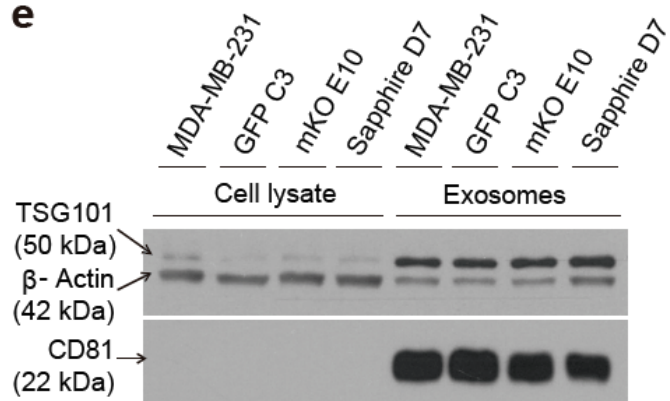

Supplement: Supplementary file 6 — Figure S5. Clone communication by exosomes. a-c. Characterization of exosomes: (a) area, (b) perimeter and (c) diameter. Significant differences were determined using unpaired t-test with Welch’s correction. Asterisks indicate significant differences when P-values are < 0.05 (*), < 0.01 (**), and < 0.001 (***). d. Representative transmission electron microscopy image of exosomes. e. Immunoblot of exosome markers (TSG101 and CD81) and housekeeping gene (β-actin). (PDF 868 kb) [file 12885_2019_5883_MOESM6_ESM.pdf]
