# Supplementary material for: The role of clonal communication and heterogeneity in breast cancer
Source: BMC Cancer. 2019 Jul 5;19:666. doi: 10.1186/s12885-019-5883-y (PMC6612119; doi:10.1186/s12885-019-5883-y)
Supplement: Supplementary file 4 — Table S1. Analysis of mKO mRNA expression vs MDA-MB-231 with the Human Cancer Metastasis Database. (PDF 511 kb) [file 12885_2019_5883_MOESM4_ESM.pdf]

Additional file 4: Table S1 Analysis of mKO mRNA expression vs MDA-MB-231 with the Human Cancer Metastasis Database

Gene	mKO vs MDA-MB-231	Evidence for role in metastasis
ADAM23	up	Intratumoral heterogeneity of ADAM23 promotes tumor growth and metastasis through LGI4 and nitric oxide signals.
ADGRG1	down	A significant correlation between GPR56, TG2, and NF-kappaB was observed that correlated with nodal metastasis and tumor invasion in esophageal squamous cell carcinoma.
AFAP1L2	down	This is the first study to reveal that XB130 overexpression may be related to the prediction of metastasis potency and poor prognosis for osteosarcoma patients.
ALPP	down	High serum alkaline phosphatase cooperating with MMP-9 is associated with metastasis in patients with primary osteosarcoma.
ANGPTL2	down	Serum ANGPTL2 improves preoperative detection of LN metastasis in CRC.
ANKRD12	down	Study revealed that ANKRD12 mRNA was downregulated in CRC tumor tissues and low ANKRD12 expression was correlated with liver metastasis and poor survival of CRC patients.
ANTXR1	down	High ANTXR1 accelerates breast tumor growth and lung metastasis.
AR	down	CCL5 signals from infiltrating bone marrow mesenchymal stem cells to HIF2alpha signals within prostate cancer (PCa) cells might play a key role in increasing PCa stem cell population and PCa metastasis via altering the androgen receptor signals.
ARL2BP	down	Alteration of EBV encoded miR-BART1 expression results in an increase in migration and invasion of nasopharyngeal carcinoma in vitro and causes metastasis in vivo. EBV-miR-BART1 directly targets the cellular tumour suppressor PTEN.
ARMC8	up	ARMc8 may enhance the invasion and metastasis of ovarian cancer cells.
BAG1	down	Expression of Bag-1 in colon cancer was closely correlated with pathologic grade, metastasis distance, Duke stage, and prognosis, but it had no effect on the pathologic type, tumor diameter, depth of invasion, and lymphoid node metastasis of the cancer.
BAIAP2	down	IRSp53 and spinophilin regulate localized Rac activation by T-lymphocyte invasion and metastasis protein 1.
BCL2	up	Survivin and Bcl-2 may act synergistically in the occurrence, development, invasion and metastasis of non-small cell lung carcinoma (NSCLC), both of which are upregulated in NSCLC tissues
BDNF	down	Higher expression of TrkB in NSCLC is closely correlated with lymph node metastasis, and BDNF probably via TrkB/Pyk2/ERK promotes the invasion of A549 cells.
BMP1	down	miR-194 suppresses metastasis of non-small cell lung cancer through regulating expression of BMP1 and p27kip1.
BMP4	down	BMP4 caused a trend towards accelerated metastasis formation, especially in bone. More work is needed to uncover the long-term effects of BMP4 and the clinical relevance of these findings.
CA9	down	This is the first evidence that CA IX may promote nasopharyngeal carcinoma metastasis.
CBLB	down	H19 non coding RNA-derived miR-675 enhances tumorigenesis and metastasis of breast cancer cells by downregulating c-Cbl and Cbl-b.
CEACAM5	down	CEA and CA19-9 provide additional diagnostic information in gastrointestinal malignancies but only CA19-9 was significantly associated with peritoneal metastasis.
CHRM3	down	Overexpression of muscarinic receptor 3 promotes metastasis in non-small-cell lung cancer.
CPE	down	N-terminal truncated carboxypeptidase E splice isoform induces tumor growth and is a biomarker for predicting future metastasis in human cancers.
CYLD	down	CYLD downregulation may promote breast cancer metastasis via NF-kappaB activation, including RANKL signaling.
CYP2J2	up	CYP2J2 has a role in cancer metastasis.
DLC1	down	Evaluation of clinical breast tumor samples revealed that reduced DLC1 expression was linked to elevated PTHLH expression and organ-specific metastasis to bone.
DOCK4	up	Activator DOCK4 as a key component of the TGF-beta/Smad pathway that promotes lung ADC cell extravasation and metastasis.
ENPP1	down	These data suggest a potential role for Enpp1 in the development of breast cancer bone metastasis.
EPSTI1	down	A novel KLF8 to EPSTI1 to VCP to NF-kappaB signaling mechanism, potentially critical for breast cancer invasion and metastasis.
ERAP1	down	The absence or downregulated expression of ERAP1 is closely related to metastasis and invasion of lymph nodes in ovarian carcinoma.
EVI5	down	Data show that heat shock transcription factor 1/miR-135b/reversion-inducing-cysteine-rich protein with kazal motifs and ecotropic viral integration site 5 axis provides novel insight into the mechanisms of hepatocellular carcinoma metastasis.
FBN1	down	FBN1 acts downstream of Aur A and BRCA2, promoting ovarian cancer metastasis through p53 and SLUG-associated signaling.
FLNA	down	FLNA expression decreased in gastric cancer and correlated significantly with lymph node metastasis, clinic stage, histological grade, and poor overall survival.
FNDC3B	down	fad104 suppressed the invasion and metastasis of melanoma cells by inhibiting activation of the STAT3 signaling pathway.
FOXO4	down	The data suggest that loss of FOXO4 expression contributes to gastric cancer growth and metastasis, and it may serve as a potential therapeutic target for gastric cancer.
FYN	down	Fyn plays an important role in prostate cancer biology by facilitating cellular growth and by regulating directed chemotaxis-a key component of metastasis.
G6PD	down	Overexpression of G6PD is associated with high risk of recurrent metastasis in primary breast carcinoma.
GCNT2	down	Hypomethylation of the GCNT2 variant 2 reflected lymph node metastasis of colorectal cancer in the tumor and normal tissues.
HK2	down	High exokinase 2 expression is associated with neuroblastoma metastasis.
IL13RA1	down	Results identified FAM120A in the IL13/IL13Ralpha2 signaling pathway as a key mediator of invasion and liver metastasis in colon cancer.
IL24	down	Suggests that IL-24 plays a profound role in suppressing tumour lymphangiogenesis, thereby reducing the likelihood of cancer metastasis via the lymphatic route.
INPP4B	down	Study provides evidence that INPP4B loss can promote follicular-like thyroid cancer progression and metastasis in the context of PTEN haploinsufficiency through the isoform-specific regulation of AKT signaling at the endosomes.
IRS2	up	This review points out that IRS-2, which is implicated in mediating signals to promote tumor cell survival, growth and motility, is a positive regulator of breast cancer metastasis.
ISG15	down	Results suggest that high ISG15 expression is an intrinsic feature of hepatocellular carcinoma and a trigger for tumorigenesis and metastasis.
JAG1	down	Notch1 and JAGD1 expression were also proven to be associated with cervical cancer invasion, lymph node metastasis, and FIGO system.
KDM4C	down	JMJD2C decreases trimethylation of histone H3 at lysine 9, and enhances HIF-1 binding to hypoxia response elements, thereby activating transcription of proteins that are required for metabolic reprogramming and for lung metastasis.
KRT7	down	Case Report: CK7+/CK20- Merkel cell carcinoma presenting as inguinal subcutaneous nodules with subsequent epidermotropic metastasis.
LIMA1	down	A major activity of DNp73 is to initiate the invasion-metastasis cascade via EPLIN-dependent IGF1R regulation.
LPAR1	down	Downregulation of EDG2 is functionally important to suppression of tumor metastasis in breast neoplasms.
LRP5	down	Lrp5 binds to Frizzled, preventing Frz-regulated non-canonical Wnt pathway activation and further non-canonical pathway-mediated tumour metastasis.
LYN	down	Diminished Lyn levels impair Claudin-2 expression in breast cancer cells. The Lyn-selective kinase inhibitor, Bafetinib (INNO-406), reduces Claudin-2 expression and suppresses breast cancer liver metastasis.
MAP1LC3B	down	High expression of LC3B, correlated with vascular invasion and lymph node metastasis, might be a novel prognostic biomarker and a potential therapy target for HCC.
MAP7D3	down	A critical role for Mdp3 in the growth and metastasis of breast cancer.
MCTS1	down	The results suggest that co-expression of CD147 and MCT1/MCT4 is related to drug resistance during EOC metastasis and could be useful therapeutic targets to prevent the development of incurable, recurrent and drug resistant EOC.
MECP2	down	The expression of 5-hmC, 5-mC, and TET2 correlated with pathologic stage, tumor grading, lymph node metastasis, and vascular thrombosis.
MITF	down	MITF expression may affect the melanoma phenotype with consequences on the survival, invasion and metastasis of melanoma cells, and a discussion of the research challenges. Review.
MYB	up	MYB is aberrantly overexpressed in PC cells and acts as a key determinant of pancreatic tumour growth and metastasis.
MYD88	down	Suggests elevated MyD88 may facilitate hepatocellular carcinoma metastasis by promoting epithelial-mesenchymal transition and tumor-initiating capabilities via PI3-K/Akt pathway.
NAT1	down	The results of this study demonstrate that NAT1 activity may be important in breast cancer growth and metastasis.

NCOA3	down	Results show that CCT-mediated AIB1 folding appears to be involved in the rigidity response of breast cancer cells, which provides novel insight into the mechanisms of bone metastasis.
NFKB2	down	DLX4 induces CD44 by stimulating IL-1beta-mediated NF-kappaB activity, thereby promoting peritoneal metastasis of ovarian cancer.
NLRP3	up	Activation of the NLRP3 inflammasome by M.hyorhinis may be associated with its promotion of gastric cancer metastasis.
NR4A2	down	NR4A2 was significantly down-regulated in synchronous liver metastasis compared with the paired gastric cancer.
PARD6A	down	Shp2 promotes metastasis of prostate cancer by attenuating the PAR3/PAR6/aPKC polarity protein complex and enhancing epithelial-to-mesenchymal transition.
PBXIP1	down	HPIP may play a critical role in oral carcinogenesis and is a potential target for anticancer therapy, with particular emphasis on its involvement in differentiation and migration/metastasis.
PDGFRB	down	Increased expression of platelet-derived growth factor receptor-beta in pancreatic cancer cells is mediated by DNA binding and structural mutants of p53, and this contributes to metastasis.
PITPNM3	down	CCL18 derived from tumor-associated macrophages (TAMs) plays a critical role in promoting breast cancer metastasis via its receptor, PITPNM3.
PLAUR	up	uPAR(+) cells injected subcutaneously into nude mice markedly increased tumor growth, induced VM formation and liver metastasis; in contrast, uPAR(-) cells did not.
PSMD10	down	Gankyrin may be functional in cervical carcinogenesis and metastasis.
PTGS2	down	COX-2 and Ki-67 are abundantly expressed in hepatolithiasis and bile duct carcinoma tissues and may play an important role in the disease occurrence, progression, and metastasis.
RAB3D	down	High expression of small GTPase Rab3D promotes cancer progression and metastasis.
RALBP1	down	Studies suggest that the expression of RalBP1 is necessary for human cancer cell metastasis, and show that the requirement for RalA expression for manifestation of this phenotype is not entirely dependent on a RalA-RalBP1 interaction.
RARRES3	down	RARRES3 downregulation engages metastasis-initiating capabilities by facilitating adhesion of the tumor cells to the lung parenchyma.
RASSF2	up	Inactivation of RASSF2A by promoter methylation correlates with lymph node metastasis in nasopharyngeal carcinoma.
RECQL	down	A novel function of RECQ1 is identified in gene regulation and indicates that RECQ1 contributes to tumor development and progression, in part, by regulating the expression of key genes that promote cancer cell migration, invasion and metastasis.
ROBO1	down	Findings indicated that Slit2/Robo1 axis could be regarded as a significant clinical parameter for predicting brain metastasis in breast cancer patients.
ROCK1	down	Data indicate that expression of ROCK1 and ROCK2 are closely associated with tumor growth and lymph node metastasis of laryngeal squamous cell carcinoma.
S100A2	down	Expression of S100A2 in cholangiocarcinoma cells significantly correlated with the histological grade, lymph node metastasis, clinical stage, and a poor survival rate of the patients. Thus it is a strong tumor marker for the cancer.
SIRT7	down	Sirt7 is overexpressed in human gastric cancers. Expression of Sirt7 is markedly correlated with tumor size, metastasis, disease stage and prognosis.
SIX1	down	SIX1 and EYA are often co-overexpressed in tumors, and the SIX1-EYA2 interaction has been shown to be critical for metastasis in a breast cancer model.
SLC2A1	down	These results indicate SLC2A1 exhibits a pivotal role in tumor growth, metastasis and glucose metabolism.
SSR4	down	Results of this study implicate TRAPD as a candidate gene with potential functions that might be associated with ultraviolet-induced melanomagenesis and metastasis.
TACSTD2	down	TROP2 overexpression and lymph node metastasis were independent prognostic markers in pulmonary adenocarcinoma.
TAP1	down	The data suggest that patients with low or defective TAP1 or calnexin in primary breast cancers may be at higher risks for developing brain metastasis due to the defects in T cell-based immunosurveillance.
TET1	down	Downregulation of TET1 due to hypermethylation is associated with breast cancer metastasis.
TGFBR1	down	High TGFBR-1 expression is associated with metastasis in non-small cell lung cancer.
TNS3	down	Tensins may represent a novel group of metastasis suppressors in the kidney, the loss of which leads to greater tumor cell motility and consequent metastasis.
TNS4	down	Cten expression is of prognostic significance in colorectal carcinoma, and delineates a Cten-ILK pathway controlling cell motility and possibly promoting metastasis.
TRAF4	down	Review highlights TGF-beta-induced SMAD-dependent signaling and non-SMAD signaling as the major pathways regulated by TRAF4 involved in breast cancer metastasis.
TRIP10	down	CIP4 promotes metastasis in triple negative breast cancer and is associated with poor prognosis.
TSPAN7	up	Loss of TSPAN7 is associated with metastasis in clear-cell renal cell carcinoma.
VSNL1	up	Distinct roles of proliferative and invasive phenotypes contributing to neuroblastoma progression which demonstrates that VSNL-1 is important in neuroblastoma metastasis.
WNT5A	up	Data suggest that WNT5A is an important molecule in promoting stem cell characteristics in NPC, leading to tumorigenesis and metastasis.
WWOX	down	HGF and TGFbeta1 of bone-metastasis microenvironment acted in coordination, influencing non-redundant pathways regulated by Twist program or Snail-transcription factor, with reversible MET switch.
XIAP	down	xIAP and cIAP1 identified as molecular targets of ceramide, and ceramide analog LCL85 shown to be an effective sensitizer in overcoming resistance of metastatic colon and breast cancers to apoptosis induction to suppress metastasis in vivo.
ZFHX3	down	ATBF1-A mRNA has a role in lymph node metastasis of breast neoplasms.
ZNF382	down	Methylation changes of GFRA1, SRF, and ZNF382 may be a potential biomarker set for prediction of gastric carcinoma metastasis.
ZNF703	down	Results show that ZNF703 expression is upregulated in colorectal cancer tissues (CRC) and is significantly correlated with tumor size, pathological grading, and serosal and lymph node metastasis suggesting that it may act as an oncogene in CRC.
